# QAPgrid: A Two Level QAP-Based Approach for Large-Scale Data Analysis and Visualization

**DOI:** 10.1371/journal.pone.0014468

**Published:** 2011-01-18

**Authors:** Mario Inostroza-Ponta, Regina Berretta, Pablo Moscato

**Affiliations:** 1 Departamento de Ingeniería Informática, Universidad de Santiago de Chile, Santiago, Chile; 2 Australian Research Centre of Excellence in Bioinformatics, Callaghan, Australia; 3 Centre for Bioinformatics, Biomarkers Discovery and Information-Based Medicine, The University of Newcastle, Callaghan, Australia; 4 Hunter Medical Research Institute, Information Based Medicine Program, John Hunter Hospital, New Lambton Heights, Australia; Dana-Farber Cancer Institute, United States of America

## Abstract

**Background:**

The visualization of large volumes of data is a computationally challenging task that often promises rewarding new insights. There is great potential in the application of new algorithms and models from combinatorial optimisation. Datasets often contain “hidden regularities” and a combined identification and visualization method should reveal these structures and present them in a way that helps analysis. While several methodologies exist, including those that use non-linear optimization algorithms, severe limitations exist even when working with only a few hundred objects.

**Methodology/Principal Findings:**

We present a new data visualization approach (QAPgrid) that reveals patterns of similarities and differences in large datasets of objects for which a similarity measure can be computed. Objects are assigned to positions on an underlying square grid in a two-dimensional space. We use the Quadratic Assignment Problem (QAP) as a mathematical model to provide an objective function for assignment of objects to positions on the grid. We employ a Memetic Algorithm (a powerful metaheuristic) to tackle the large instances of this NP-hard combinatorial optimization problem, and we show its performance on the visualization of real data sets.

**Conclusions/Significance:**

Overall, the results show that QAPgrid algorithm is able to produce a layout that represents the relationships between objects in the data set. Furthermore, it also represents the relationships between clusters that are feed into the algorithm. We apply the QAPgrid on the 84 Indo-European languages instance, producing a near-optimal layout. Next, we produce a layout of 470 world universities with an observed high degree of correlation with the score used by the *Academic Ranking of World Universities* compiled in the *The Shanghai Jiao Tong University Academic Ranking of World Universities* without the need of an *ad hoc* weighting of attributes. Finally, our Gene Ontology-based study on *Saccharomyces cerevisiae* fully demonstrates the scalability and precision of our method as a novel alternative tool for functional genomics.

## Introduction

One of the existing alternatives for the visualization of the relationships among objects is to organize them in a grid layout. Consider a two dimensional square grid of 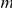 locations and a set of 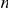 objects. The basic idea is to assign the given set of 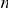 objects (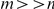) to positions in the grid, such that the final result conveys information about the similarity relationship among the objects. This alternative has not been sufficiently explored and exploited to its full potential and our contribution aims at closing this gap. Ideally, if two objects are highly similar, they should be located in nearby locations in the grid. The similarity relationship among objects is addressed by first defining a distance matrix. Accordingly, highly similar objects have a small distance and viceversa.

A grid layout has been employed by Li and Kurata [1] for the visualization of biochemical networks. They proposed a method that produces a grid layout of a given biochemical network based on the graph representation of the network. The authors define a weight matrix such that closely related nodes receive positive weights and weakly related nodes receive negative weights. The benefit of this approach is compared against an alternative force-directed graph layout algorithm. The latter produces a good visualization of the structure of the graph, but unlike our method, the functional modules are not well represented. A more general method for data visualization using a grid layout is presented by Abbiw-Jackson et al. [2]. The authors show a divide and conquer algorithm that first distributes the data in a 2×2 grid, and then iteratively divides each of the grid positions in a new 2×2 grid. The method is fast, accurate and represents the original distances. However, the main disadvantage is that does not guarantee that every object of the data set will be placed in a different cell of the grid, resulting in a somewhat cluttered visualization of the objects. In bioinformatics, a well known tool for graph visualization is Cytoscape [3]. This tool has incorporated several graph drawing algorithms (i.e. force-directed, tree, cyclic, organic, among others). It is mainly used as a bioinformatic software platform for visualizing molecular interaction networks. Due to its popularity and wide acceptance, the force-directed algorithm implemented in this tool will be used to compare the output of our proposed solution. Alternative tools for graph visualization also used in the scientific community include Ondex [4] and yEd [5], which also implement several graph layout algorithms.

In this paper we present results of an approach for data visualization using a grid layout that employs the Quadratic Assignment Problem (QAP) as the base mathematical model. The QAP is a well-known combinatorial optimization problem with the goal of assigning objects to locations, considering the flow between objects and the distance between locations. Since the QAP belongs to the NP-class, we apply a Memetic Algorithm (a very sucessful metaheuristic, for reviews on the field see [6]–[8]) to tackle these QAP instances. In order to demonstrate the benefits of our approach for data visualization, we use three different datasets: first, a dataset of distances between 84 Indo-European Languages composed of several language families; second, a dataset with the ranking information for the 470 world-best universities during three consecutive years; and third, a dataset with the gene expressions of more than 2,400 gene probes of *Saccharomyces Cerevisiae* under several experiments. In all the three cases, our algorithm was able to produce a visualization representing the languages families, the performance of the universities and also the gene expression similarities (including similarities using the gene ontologies terms of genes). In particular, the algorithm was able to put in closer locations clusters that share highly similar objects.

## Methods

### Data visualization using Quadratic Assignment Problem

The Quadratic Assignment Problem (QAP) is a well known combinatorial optimization problem [9]–[11], in which the task is to assign each one of a set of 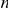 objects to 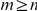 locations. No two objects can be assigned to the same location. Part of the problem definition is a flow matrix between each pair of objects 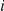 and 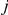 (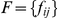) and the distance between each pair of locations 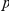 and 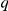 (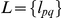) are given as input. The objective is to minimize the cost function given by Eq. (1). Let 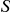 be a solution for the QAP, where 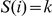 means that object 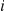 (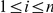) is assigned to location 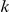 (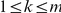).
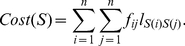
(1)


The QAP was first presented as a mathematical model related to economic activities by Koopmans and Berkmann [12]. After that initial contribution to the literature, different applications of the QAP have been presented including: campus building layout [13], hospital planning [14], placement of electronic components [15], [16], among others. The QAP was proved to belong to the NP-Hard class of problems by Sahni and González [17]. An exact solution of the QAP can only be found for instances of size up to 30 objects in a reasonable computational time. As a consequence, several metaheuristics have been proposed, such as Genetic Algorithms [18]–[21], Ant Colonies [22], [23], Memetic Algorithms [9], [24], [25], Tabu Search [26]–[28], among others. We applied an ad hoc Memetic Algorithm to solve the large instances of this contribution.

### Creating QAP instances for data visualization

In order to create an instance of the QAP to solve the visualization problem, we need to define the matrices 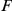 and 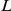. Let 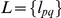 be the Euclidean distance between locations 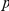 and 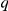 on a grid with 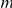 locations. Let 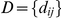 be the distance between objects 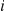 and 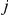. We define 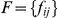 as follows.
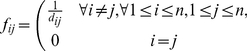
(2)


As a result, objects that have a small distance between them will have a large flow and consequently, a good solution for the QAP will assign similar objects in nearby locations.

If we have a pre-determined partition of the set of objects (clusters), we can also consider each cluster (a set of objects) as an single object and find a best layout for the clusters as well. In this case, we can define another QAP instance (for this top-level layout problem). The layout of clusters would then be based on the similarity relationship between members of the clusters. Consider 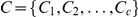 a set of 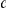 disjoint groups representing 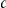 clusters. The matrix 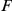 for the layout of clusters is defined as Eq.(3), where 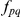 represents the flow between objects 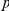 and 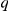 as defined in Eq.(2). It is expected that clusters that have strong similarities between their objects will be assigned closer in the final layout. The QAP, as a mathematical model for this requirement, embodies our quest that the final layout should globally have this property.
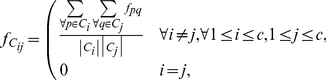
(3)


If we have as input, a set of objects and a set of clusters, the layout of objects can be obtained in two steps: first we produce the layout of the objects in each cluster (using the 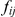 values as defined in (2)), and then we compute a layout of the clusters (now using 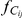 defined in (3)).

### Integrating the information given by a proximity graph

We will now show that our QAPgrip is also able to naturally incorporate other sources of information. In some cases, we may have a *proximity graph*, indicating that some adjacency preferences in the final layout should be enforced. In a proximity graph there is an edge between two vertices if they fulfill a certain proximity condition. Examples of well known proximity graphs are Minimum Spanning Tree, k-Nearest Neighbor, and the Relative Neighborhood Graph. In order to incorporate the proximity graph (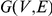, 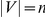) in our approach, the definition of flows between objects defined in (2) can be modified as follows:
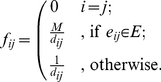
(4)


The parameter 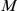 allows to arbitrarily increase the flow between pairs of objects that have an edge in the proximity graph. This will result that those objects will have an even higher flow, so we “enforce” the requirement to lay them closer in the final solution of the QAP instance and allows users to explore different layouts. The effect of the parameter 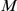 in the grid layout result is shown later.

### A Memetic algorithm for the Quadratic Assignment Problem

Memetic Algorithms (MAs) is a denomination for a class of population based search strategy that has proven to be of extreme practical success in a variety of applications [6]–[8], [29]–[32]. We have extensive experience in the developement of methods using MAs and other metaheuristics to tackle complex combinatorial optimization problems (see [6]).

In this work we implement a MA with similar elements employed before [30], [31], [33]–[35]. It uses a hierarchically structured population organized as a ternary tree and different local searches and incorporates Tabu Search ([36]). For recombination, we use a modified version of the *cycle crossover* also used by Merz and Freisleben [25]. In addition, extra strategies are applied to deal with premature convergence. In the supplementary material (File S1), we describe in details the MA implemented.

## Results

The QAPgrid was written in Java 1.6 and the computational tests were run on a computer with Intel Core i3 CPU (1.86 Ghz, 2GB RAM). We used three different datasets to perform computational tests: (1) a dataset of 84 Indo-European languages, (2) a dataset containing information about the ranking of 470 world class universities and (3) a dataset with the gene expression of 2,467 genes of *Saccharomyces cerevisiae*. Table 1 shows the average computational times applying QAPgrid with the Memetic Algorithm running for 10 iterations.

**Table 1:** Execution time on the test instances.

Instance	# objects	# clusters	Largest cluster	Time (sec)
Languages	84	17	13	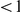
Universities	470	24	70	12
Yeast samples	79	9	19	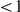
Yeast genes	2,467	52	592	2300

The time shown corresponds to the average time of 10 iterations. The largest cluster column shows the largest instance that the QAPgrid algorithm needs to solve. However, it is also possible that the largest instance corresponds to the instance created to produce the layout of clusters. This situation occurs for the Languages data set, where the instance for the layout of clusters has 17 objects.

The algorithm returns a solution as a GML file which contains the location of each individual object in the grid. In order to visualize the resulting GML file with the final layout produced by our algorithm we use the freely available package *yEd* (http://www.yworks.com/en/products_yed_about.html).

### Indo-European languages data set

First we use a data set that contains the distance matrix for 84 Indo-European Languages from 9 families, generated by Dyen, Kruskal and Black [37] (http://www.ntu.edu.au/education/langs/ielex/IE-DATA1). The distances are based on the mean percent difference in cognacy, using the 200 Swadesh words. It is expected that languages from the same family will be kept close in the layout produced by QAPgrid. This highly regarded dataset was also used by Bryant, Filimon and Gray [38] to study the performance of two algorithms for building phylogenetic networks.

The experiments and results are presented in two phases. First, we show the results of QAPgrid integrating as graph structures the results of Minimum Spanning Tree (MST) and k-Nearest-Neighbours (kNN). Consequently, 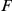 and 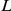 are defined using (4) and the Euclidean distance between the positions in the grid, respectively. Note that for 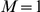, (4) is equivalent of (2). Next, we integrate a clustering algorithm result using the MSTkNN algorithm, explained below. In this case, QAPgrid first decides the layout of the objects in each cluster (using 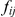 defined in (2)), and then the layout of the clusters (using 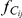 defined in (3)).

### Integrating with a proximity graph

**Figure 1:**
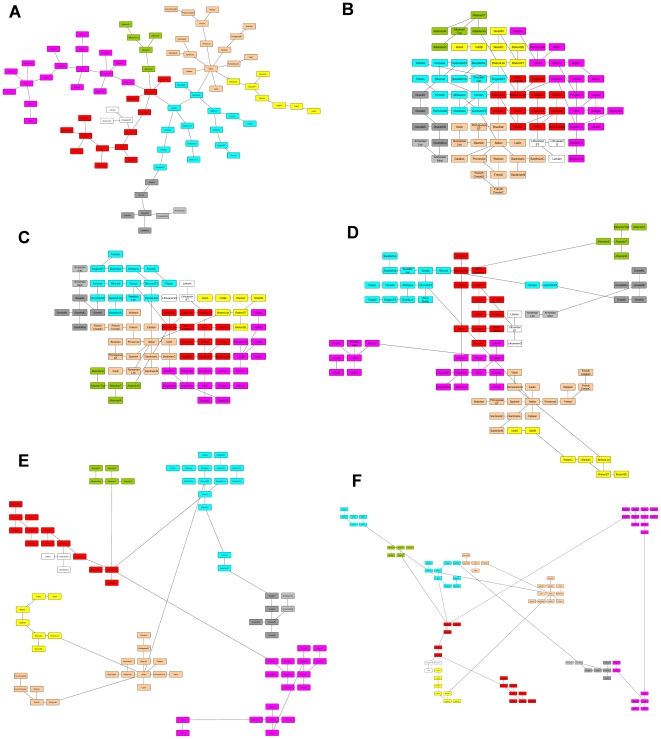
Effect of the parameter 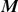 which controls a user-defined enforcing constraint to preserve adjacency relationships. Figure (A) shows the layout produced by the force-directed algorithm implemented by the public domain software yEd using the Minimum Spanning Tree (MST) on the 84 Indo-European Languages dataset. The MST is a proximity graph and we aim at producing layouts that preserve most of the adjacency relationships (edges in the MST). Figures (B) to (F) present the result of the layout parameterized by different values of 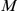: 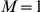
(B), 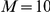
(C), 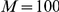
(D), 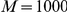
(E), and 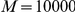
(F). In yellow are the Celtic languages, in light brown are the Romance languages, Greek-Armenian languages are colored in gray, Baltic languages in white, Germanic languages in light blue, Slavic in red, and the Indo-Iranian languages in purple.

In order to integrate a graph structure, we first compute the Minimum Spanning Tree using the distance matrix of the Indo-European languages. Then, we create instances of QAP using 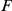 and 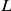 defined by (4) with different 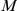 values. Figure 1 shows the results for 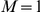 (Figure 1B), 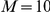 (Figure 1C), 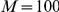 (Figure 1D), 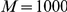 (Figure 1E) and 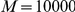 (Figure 1F), where each color represents languages from the same family. The effect of the parameter 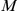 is clear; it helps to place objects connected by an edge closer while arranging the rest of the objects according to their flows. When 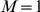, no relationship is more important than other. When 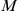 value increases, we start to see the groups more clearly. Figure 1A shows the results of the force-directed graph drawing algorithm implemented in yEd on the same MST. The structure of the graph is well represented, although, the drawing does not give any other information about how the objects are related.

**Figure 2:**
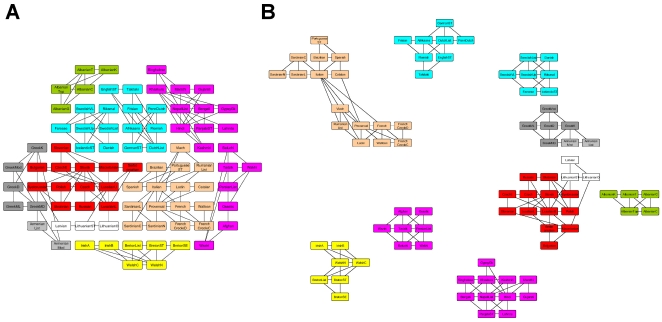
Effect of factor 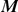 in the grid layout of the 3-NN graph. In this case, we have used the 3-nearest neighbour graph as a proximity graph whose adjacency relationships need to be preserved. The pictures show the integration of the 3 nearest neighbor graph information in the grid layout process. The graph contains clusters that are fully represented in the final layout when the algorithms uses a factor of 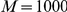 (B), but not when it uses 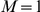 (A). In (B) the Greek-Armenian languages (in gray) constitute a separated subgraph and the same happens with the Celtic languages (in yellow). Also in (B) the Germanic languages (in light blue) are now naturally separated in North Germanic and West Germanic groups, and the Indic and Iranian groups are now separated.

The effect is even more clear when we use 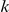-Nearest Neighbors as the proximity graph. In Figure 2 we show the results using *k*-NN with 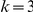, 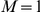 and 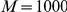. The layout is able to identify the groups presented in the graph, while at the same time, arrange the objects according to the overall flow of the system.

### Integrating cluster information

In this experiment, we use the clustering algorithm *MSTkNN*. Basically, *MSTkNN* is a graph based clustering algorithm that constructs a disconnected graph by computing the intersection of the edge sets of the Minimum Spanning Tree and k-Nearest Neighbors proximity graphs. It automatically computes the number of nearest neighbors 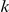 to consider for each cluster according to the number of vertices in it. It has been successfully used in a stock market dataset [39], a gene expression dataset [40] and in a prostate cancer trial dataset [41].

**Figure 3:**
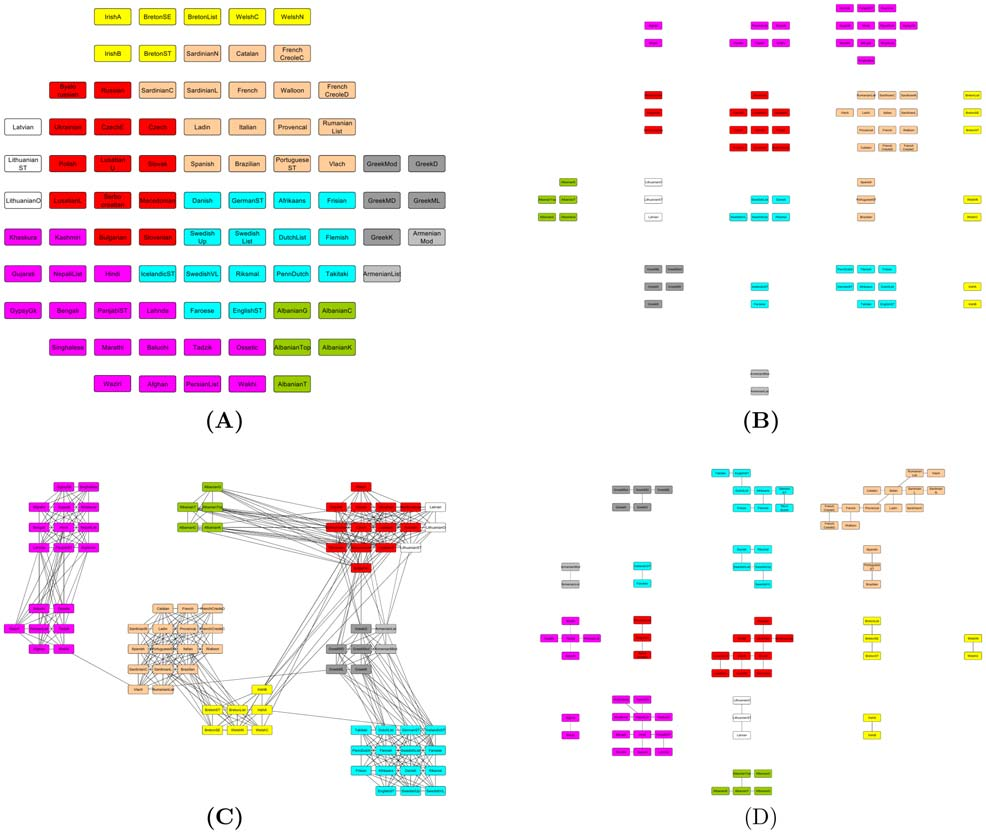
Result of QAPgrid on the Indo-European languages. The figure shows: (A) the layout only considering the distances between languages, (B) incorporating the result of the MSTkNN cluster algorithm, (C) incorporating the result of the (k = 10)-NN graph and (D) incorporating the result of the MSTkNN cluster algorithm and the MST on each cluster.

Figure 3 shows the layout produced after running the QAPgrid for 10 iterations with different scenarios. Figure 3A uses the original data set (it does not consider a graph structure or clustering of the objects). Figure 3B incorporates the result of the *MSTkNN* clustering algorithm. Consequently, after applying the *MSTkNN* clustering algorithm, we solved a QAP instance for the subset of objects in each cluster and a final QAP instance, where each cluster is considered as an object, using Eq.(3) to define the matrix 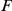. Next, Figure 3C integrates the 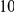-nearest neighbor graph structure. Finally, Figure 3D incorporates the result of the *MSTkNN* clustering algorithm and a Minimum Spanning Tree structure for the objects of each cluster.

It is possible to see that the integration of the result of the *MSTkNN* clustering algorithm makes more clear the organization of the objects, keeping objects from the same language family closer. The clusters are composed of languages within the same family that are also located closer. Figure 3 shows the potential of our model to produce a grid layout of a dataset represented by a distance matrix and to integrate in the process the information given by clustering algorithm and/or a graph.

**Figure 4:**
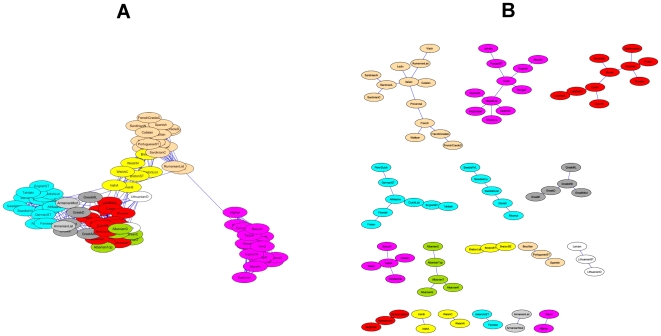
A force-directed layout using Cytoscape [3]. The figure shows the result of a force directed layout algorithm on a popular software package used in bioinformatics. Figure (A) uses the 10 nearest neighbor graph (and should be compared with Figure 3C) and (B) shows the result when we use the MST (and could be compared with that of Figure 3B). In (A) the layout is not clear and it only shows part of the graph structure. In (B) the structure of the MST is represented, but the location of the clusters in respect to each other is not guided by inter cluster similarities so the final layout misses important relationships. The color coding of these languages helps us to illustrate that our QAPgrid allows us to preserve important similarities between the clusters.

The results of an alternative and widely used approach is shown in Figure 4. In this figure the layouts are produced by the force-directed algorithm implemented in Cytoscape [3] for two different graphs. The force-directed layout algorithm is a widely used alternative in the scientific community and it considers only the edges of the input graph. We can see that the layouts are driven only by the graph structure, since there is no more information that we can utilise, unless we modify the graph structure. In Figure 4A, the graph only provides a separation of the Indo-Iranian languages, but there is no information about the rest. The clusters in Figure 4B are organized according to the size, and only to produce a visual representation of them. This situation does not happen using QAPgrid (Figure 3C) where the clusters are organized according to the relationships of their members.

### The Shanghai Jiao Tong University Academic Ranking of World Universities dataset

The ranking of universities has become a common task performed by many institutions, each of them proposes a different ranking based in several weighted categories. Examples of those rankings are: *Webometrics Ranking of World Universities* (http://www.webometrics.info/index.html), *THES - QS World Universities Rankings* (http://www.thes.co.uk/worldrankings) and *Academic Ranking of World Universities* (http://www.arwu.org/ranking.htm) (*ARWU*). The first ranking measures the visibility of the universities and their global performance in the web. The last two attempt to measure the performance of the universities based in categories like prizes received by members, citations, and publications. The ARWU is generally considered one of the more respectable attempts to measure quality and is widely recognized. The THES ranking has been widely criticized because of its excessive emphasis on peer review (see for instance [42]), that would highly favour British universities.

**Figure 5:**
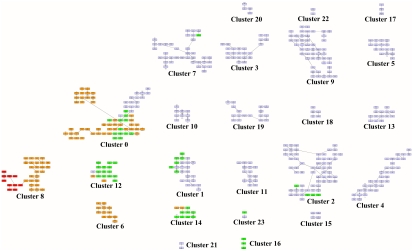
A layout of the 24 clusters found by QAPgrid using as extra input the result of the MSTkNN algorithm on the Shanghai Jiao Tong University Academic Ranking of World Universities dataset. Thanks to a measure of similarity that we have computed for each pair of clusters, followed by our optimization of the layout position of each cluster, we have a layout that reflects the academic standards of the most highly ranked 470 universities in the world. The final arrangement shows a distinctive group of the best universities in the left side (Cluster 8), that contains the top 10 universities according to the Shanghai Jiao Tong University ranking. We also note that there is a correlation between their ranking method and the final position in the layout as highlighted by the color coding scheme. This is important to highlight, as our method is entirely data-driven, and not biased by ad hoc weights for each attribute to produce a ranking. We note that some high performing universities (like University of Paris 05, University of Paris 07, and University of Paris 09, University of Vienna, The Technion, etc.,) and others at lower positions in Shanghai Jiao Tong's ranking nevertheless they share many similar characteristics with other universities in Cluster 0 (a group that appears as being led by other members like the Imperial College London, University College London, Kyoto University and University of Toronto). The university of Queensland, in Cluster 7, is the only highly ranked university in Shanghai Jiao Tong University Academic ranking that is in that cluster. This is a group of 38 highly productive institutions that include peer universities like Tsing-Hua University and National Taiwan University, just to mention two of them, that appear to yet have not scored well in the Award category, which is apparently a strong requirement to “jump” to a higher-performing cluster (Cluster 0, for instance). To understand better these results, we recommend the reader to check these clusters with the aggregated performance profile for each cluster of universities in Figure 6.

Here we use the available data for the ARWU ranking for years 2004 to 2006. Only those universities presented in these three consecutive years were considered. Each university is then described by an array of 18 values, that represent the scores of each of the 6 categories in the three years. The distance between universities is computed using the Euclidean distance measure. Initially, we applied the *MSTkNN* clustering algorithm which identified 24 clusters of different sizes. We then applied the QAPgrid algorithm producing the layout shown in Figure 5. In order to see if the original ranking is reflected in the final layout, the universities are colored according to the ranking: top 10 in red; those in the top 100 (but not in the top 10) are colored in orange; top 150 (not in the top 100) are colored in green and the rest in light blue (a larger version of the figure can be found in the supplementary materials - Files S2 and S3, together with the list of the members of each cluster).

The QAPgrid layout is in a partial agreement with the ARWU ranking aggregated score, without the need of arbitrary weights for each university attribute as it is required for the ARWU methodology. More specifically, the top 10 universities (according to the ARWU ranking) are in the same cluster (Cluster 8), together with other universities in the top 100 list of the ARWU. There are several other universities that are close to that high-performance cluster. Near to Cluster 8 (note that the labelling is arbitrary and does not correspond with a putative ranking) we can find smaller clusters composed of universities which are among the top 150 according to the ARWU ranking. Near to this group we can find Cluster 21 which is only composed of two universities (*Seoul National University* and *University of Sao Paulo*). We note the color coding we have used; they are not in the top 150 according to the ARWU ranking. However, *University of Sao Paulo* is indeed among the top 150 for years 2005 and 2006, and also *Seoul National University* is in the top 150 in year 2005 and they seem to share a very similar pattern of performance indicators. Then, the layout is showing the close relationship in the performance of the two universities with the top 150.

**Figure 6:**
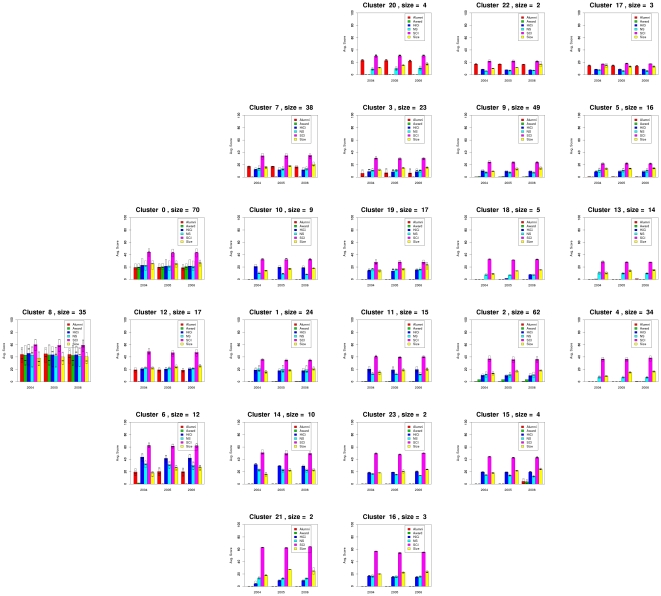
Aggregated performance profiles of the universities in each cluster using the same layout as that of Figure 5. This figure shows the profile of each one of the clusters found by MSTkNN and clearly demonstrates the ability of QAPgrid to locate together clusters with similar profiles across the three consecutive years of this study. For each year the score for Alumni, Award, HiCi, NS, SCI and Size of the components of the cluster is shown. For each category we also include a box representing the first and third quartile. The best universities are located in clusters towards the left marging of the figure. We appreciate the different “avenues” to academic excellence. Some institutions (see for instance Cluster 21) are highly productive in terms of their SCI results but still lack behind in the Alumni, Award, and HiCi rankings. As a consequence of the layout and the analysis of this figure, we have recognized the strong profile of the group of universities in Cluster 6 (a group that includes Duke University, Boston University, McGill University, etc.) and other which were perhaps disadvantaged by the weighting scheme in Shanghai Jiao Tong University Ranking scheme (like Tohoku University, in Cluster 12) that shares a position in this cluster with peers Osaka University and Tokyo Institute of Technlogy. We refer the reader to the comprehensive online supplementary material to investigate the location of individual universities (File S2).

Figure 6 shows the profiles of each cluster of universities. For each cluster, the figure shows the average value for the clusters' members on each of the six categories in each of the three years considered in the experiment. For each category it shows the median value and a box representing the first and third quartile as well. The location of the graphs correspond with the same location of the respective clusters in Figure 5.

From Figure 6 it is easy to see that:

1. Clusters are indeed composed of universities that have a similar performance across the three years.
2. Clusters with similar profiles are located closer in the layout, with the best universities in the clusters located towards the left bottom of the figure.

We observe that Cluster 8 has the “top ten” universities, the group of the ten most performing universities according to the ARWU score. However, Cluster 8 contains another group of universities (in orange) which are also well ranked including Yale University (ranked 11-11-11 in the ARWU rankings for 2004, 2005, and 2006 respectively), Carnegie Mellon University (ranked 62-54-56), Cornell University (ranked 12-12-12), the Techonological Univeristy of Munich (ranked 27-27-27), among others.

A different group, Cluster 0, reveals a highly heterogeneous set of universities which are not highly ranked according to the ARWU, nevertheless they are among the high performance group of univerisites, such as Imperial College of London, University of Edinburgh and University of Toronto (in orange), University of Liverpool, University of Pisa and University of Frankfurt (in green), and University of Vienna, Universidad de Buenos Aires and University of Paris V (in light blue). Also, Cluster 12 contains a relatively homogeneous cluster (most of them are green), of less performing universities than the ones in Clusters 0 and Cluster 6. Particularly, the profile of Cluster 0 and Cluster 12 are similar except that Cluster 12 does not perform well in the Award category (Staff of an institution winning Nobel Prize or Field Medals). Interestingly, two clusters near the bottom of the Figure 6 (Cluster 21-Seoul National University and University of Sao Paulo- and Cluster 16-Hokkaido University, Kyushu University and National University of Singapore) indicate highly productive universities in one particular category (SCI), but less in others (HiCi, NS and Size) and nil performance in Alumni and Award. Overall this reveals that our integrated layout has produced a visualization that has a high correlation with the ARWU score, but also reveals other correlations in the complete performance profile, without the need of an arbitrary selection of weights to create such an ad hoc score (removing a subjective parametrization that is always highly controversial).

**Figure 7:**
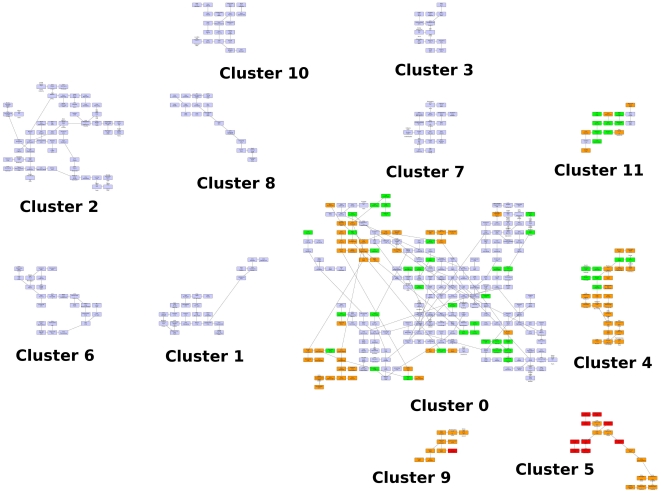
A layout of the 12 clusters found by QAPgrid using as extra input the result of the MSTkNN algorithm on the Shanghai Jiao Tong University Academic Ranking of World Universities dataset removing the categories Alumni and Award.

**Figure 8:**
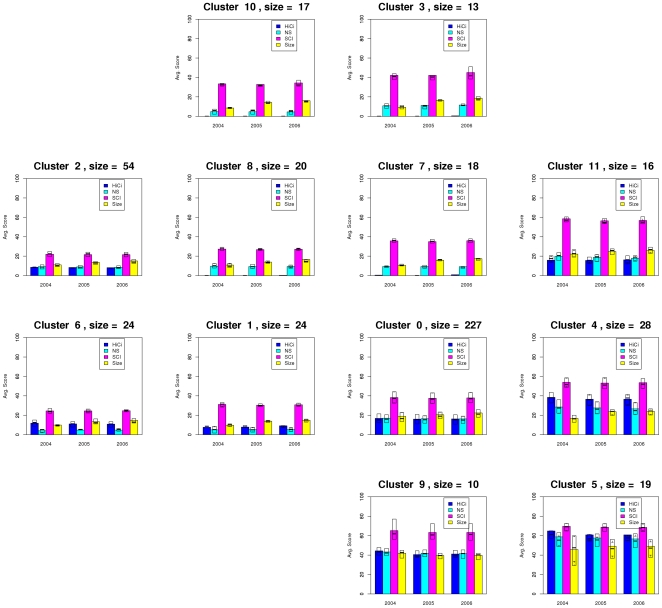
Aggregated performance profiles of the universities in each cluster using the same layout as that of Figure 7.

An additional experiment was carried out in order to analyze the result if the categories Alumni and Award are removed from the dataset in each of the years. Then, each university is represented by an array of 12 values, and the distance between them is also computed using the Euclidean distance. In Figure 7 we show the layout obtained after the application of the QAPgrid algorithm, incorporating the clustering generated by MSTkNN. The profile of each cluster is shown in Figure 8. We obtain 12 clusters, with the largest one composed by 227 universities. Overall, from the figure it is possible to appreciate a similar behaviour to the original dataset, with best performing universities assigned to nearby locations, but there are some other interesting facts. First, members of 11 clusters were still together in the new partition, either in separate clusters or joined with members of other clusters. However, most of the high performance clusters were separated. For example, members of cluster 8 from Figure 5 were separated in three groups, with one of them containing all but one (Univ. Chicago) of the top ten universities according to the ARWU ranking (cluster 5 in the new result). Univ. Chicago is now with cluster 9, which has other 9 top 100 universities (Imperial Coll London, Univ. Toronto, Duke Univ, among others). Cluster 21 from Figure 5 (Seoul Nat Univ. and Univ. Sao Paulo) are now together with other 14 best performing universities in cluster 11 from Figure 7 (Moscow state Univ., Univ. Melbourne, Univ. Sydney, among others). This confirms the fact that in the first experiment the algorithm located cluster 21 close to other well performing clusters. Some other universities, now deprived of counting Alumni and Award as proxy for quality, have moved to other clusters. For instance, University of Buenos Aires now in Cluster 7, leaving the proximity that shared with other highly ARWU-ranked universities in the previous experiment.

Figure 5 and Figure 6 show the potential of QAPgrid to produce a layout that represents the relationships between the objects in the dataset. In this particular experiment, the layout represents the relationships in the performance of universities. Here we combine the visualization benefits of QAPgrid with the benefits of the clustering algorithm MSTkNN that we have previously applied to a stock market dataset [39], a yeast gene expression dataset [40] and a prostate cancer trial dataset [41].

### Visualizing genes correlated expression patterns

Our final experiment illustrates how the QAPgrid yields novel insights on the functional genomic of yeast as inferred from that gene expression clustering results. The yeast *Saccharomyces cerevisiae* is among the most researched microorganisms. The arrival of microarray technologies made possible the analysis and understanding of the genes' behaviour in the different cell cycles stages of the budding yeast. One of the first works on the analysis of a gene expression microarray dataset for yeast was presented by Eisen et al. [43], which is among the most cited works in microarray of the *S*accharomyces cerevisiae (a highly cited contribution as reported by Google Scholar and ISI Web of knowledge figures of 10,054 and 6,963, respectively). We then turned our attention to this dataset as a large number of methods have been applied on it and is considered a benchmark in the development of algorithms for bioinformatics.

Here we present the results obtained with QAPgrid using the freely available dataset corresponding to the second figure of the original work presented in [43]. This dataset is composed of the expression of 2,467 genes on 79 samples corresponding to 8 different experiments of the budding yeast: *alpha factor* (18 samples), *cdc15* (15 samples), *cold shock* (4 samples), *diauxic shift* (7 samples), *DTT shock* (4 samples), *elutriation* (14 samples), *heat shock* (6 samples) and *sporulation* (11 samples).

In order to show the versatility of the method we perform two tests on the dataset. We first apply the method to the task of producing a visualization of the set of samples, with the assumption that samples from the same experiments should be clustered together so they should be closely located in the final layout produced by QAPgrid. Second, we apply the algorithm on genes, expecting to observe that genes with a similar function will be clustered and/or laying together. This was one of the main findings of the original publication: “*We have found in the budding yeast Saccharomyces cerevisiae that clustering gene expression data groups together efficiently genes of known similar function*” [43].

### Layout of samples

To analyze the samples we first apply the QAPgrid on the 79 samples considering only the distance matrix computed between each pair of samples. Then, we use the result of the MSTkNN clustering algorithm [40] to produce the integrated clustering/layout of the 79 samples.

**Figure 9:**
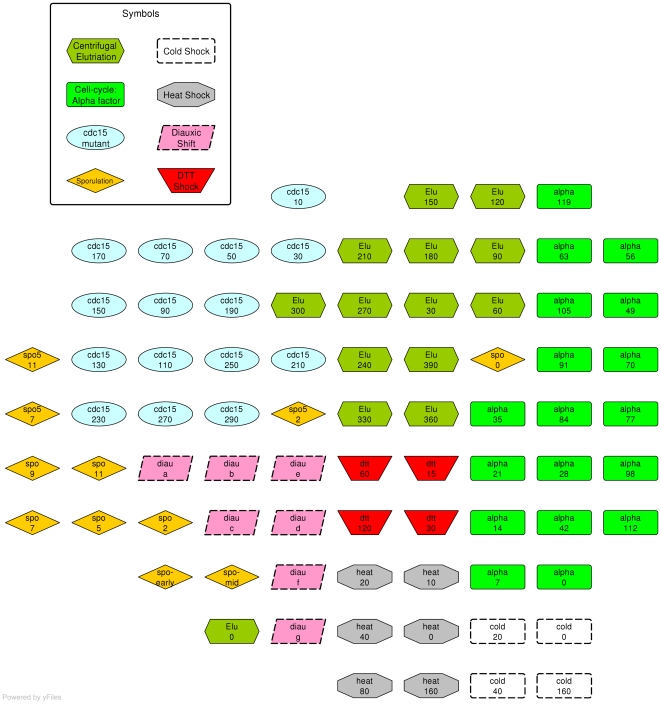
Final layout produced by QAPgrid of the 79 samples of Eisen's yeast dataset [43]. The samples were colored after the layout was obtained according to the experiment in which they belong. We can see that the samples location correlate well with the experiment to which they belong, with the obvious exception of some of them due to the fact that they are initial/early states. We refer to Figure 10 and Table 2 for related results.

Each sample is represented by the expression of the 2,467 genes. The distance between two samples is computed using the Pearson Correlation based distance (5). We run QAPgrid on the samples for 10 iterations. Once the layout is obtained, each sample is colored according to the experiment to which it belongs (see Figure 9). It is clear to see that the samples are mainly organized according to the experiment to which they belong. However, the layout shows three samples that seem to be “misplaced”: *spo_0*, *spo5_2* and *elu_0*, though two of them correspond to initial values.
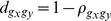
(5)


**Figure 10:**
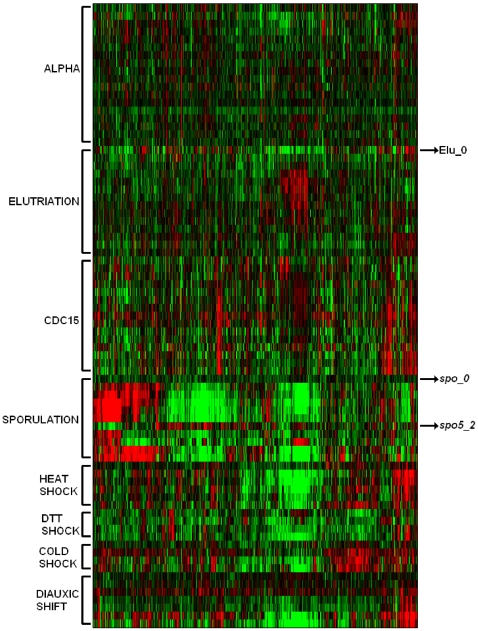
A traditional heatmap representation of the expression of the 79 samples on the 2,467 genes. Each group of samples is separated according to the experiment ordered using the Memetic Algorithm described in Ref. [31]. It is clear to see that the expression of those samples is very different from the others in the group that has the same label.

The gene expression patterns of those samples help us to understand the result. In Table 2 we show the average distance of the samples to the surrounding groups of samples and its closest sample. We see for each of the “misplaced” samples that the correspondent group is not the closest one, and furthermore, the closest sample does not belong to their group. The heatmap in Figure 10 shows the expression of each of the samples ordered by experiment across of all genes. It is clear the difference in the expression of samples *spo_0*, *spo5_2* and *elu_0* with their corresponding labelled group's.

**Table 2:** Nearest neighbors of misplaced samples in yeast data set.

Sample	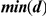	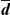 to	
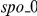	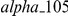	sporulation	1.009
		alpha	0.887
		elutriation	0.765
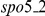	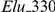	sporulation	1.247
		elutriation	0.879
		cdc-15	0.981
		diauxic shift	0.853
		DTT shock	0.881
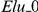	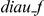	elutriation	1.041
		sporulation	0.800
		diauxic shift	0.981

Closest sample and group for each of the “misplaced” samples in the sample layout of Figure 9.

**Figure 11:**
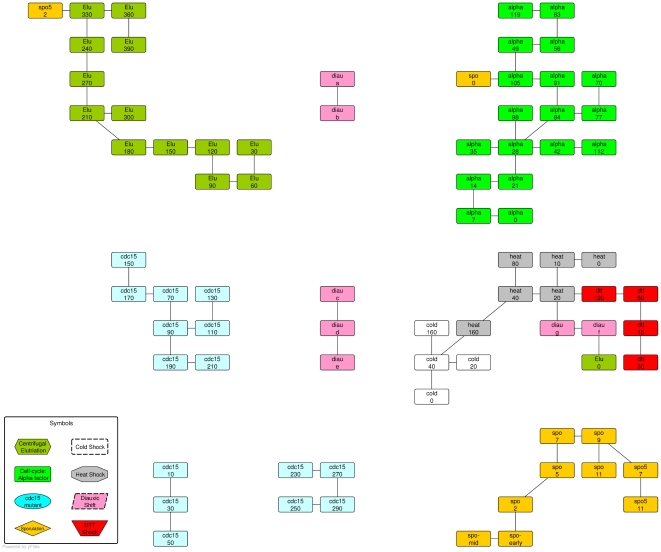
Final layout of the clusters of yeast samples. The 79 samples of Eisen's yeast dataset using the same coloring scheme. The clusters have been produced using the MSTkNN algorithm.

In the next experiment we use MSTkNN clustering algorithm on samples and the result was used by QAPgrid. In Figure 11 we show the result of the layout of the clusters and the cluster's components. QAPgrid puts close those groups of samples from the same experiment that were split by MSTkNN (*cdc15* and *diauxic* samples). In addition, in the cluster with mixed samples, they are organized mainly by the experiment. This reproduces what we observed in Figure 9.

### Layout of genes

**Figure 12:**
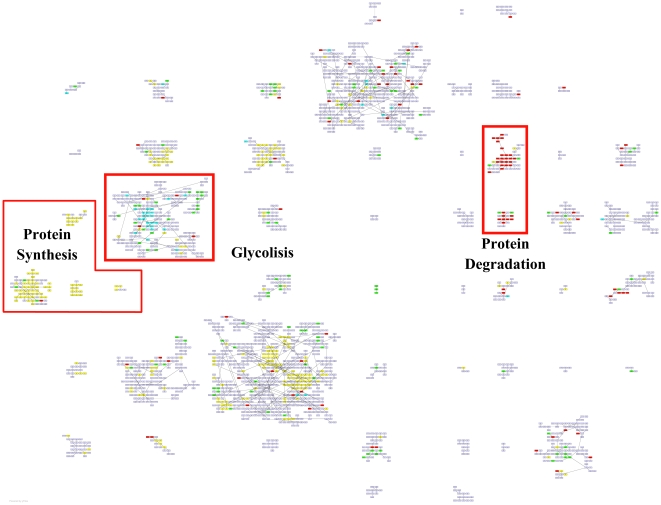
Grid layout of the 2,467 gene probes of Eisen's yeast dataset. Three biological processes are highlighted: *Glycolysis*, *Protein Synthesis* and *Protein Degradation*. Overall, the genes are mainly located according to their biological function, even when they were assigned to different clusters (i.e. *Protein Synthesis* and *Protein Degradation*). As expected, sometimes probes for genes that share a GO term annotation are located in different clusters, but due to the global optimization nature of QAPgrid, these clusters tend to be close together in the final layout.

We now aim to verify if the genes with similar biological function are kept close in a layout produced by QAPgrid. We first concentrate the discussion on the three main biological functions that were also discussed in the original publication: *Protein Synthesis*, *Glycolysis* and *Protein Degradation*. In Figure 12 we show the layout obtained by QAPgrid. Using the information provided in the supplementary material website (http://www.pnas.org/cgi/content/full/95/25/14863/DC1) of the original publication we highlight the places with a high concentration of genes with the same biological function.

Both the clustering and the layout of genes are clearly driven by the gene expression patterns and they seem to correlate well with the biological function. Where the clustering algorithm was too sensitive and separated a group of biologically-related genes, QAPgrid is nevertheless able to put the respective clusters closer in the final assignment. Again, this seems to validate the notion that similar patterns of gene expression correlate with similar biological functions in these type of studies. This situation happens for clusters which are mainly composed of genes related with the biological function of *protein synthesis* and *protein degradation* as indicated in Figure 12. Furthermore, QAPgrid organizes the genes related to *Glycolysis* closer in the layout of the respective cluster.

In order to further analyze the results with more updated information, we use the Gene Ontology Term Finder [44] to find the biological processes involved in all the clusters. The website allows searching for significant shared GO terms used to describe a provided list of genes, with the aim to help the discovery of commonalities between the genes in the list. We use the whole set of 2,467 genes as a background for comparative purposes.

**Figure 13:**
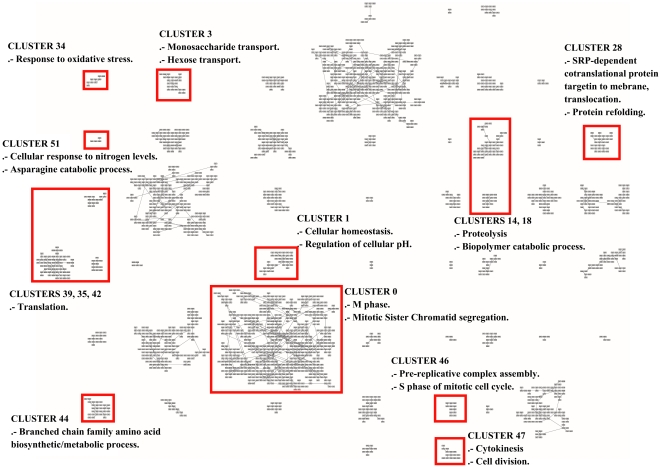
Grid layout of the 2,467 gene probes of Eisen's yeast dataset. In this Figure, as well as in Figures 14 and 15, we highlight a GO term which has been uniquely and statistically well-associated to a single particular cluster. In such a way, we highlight the unique biological annotation associated to each clusters.

**Figure 14:**
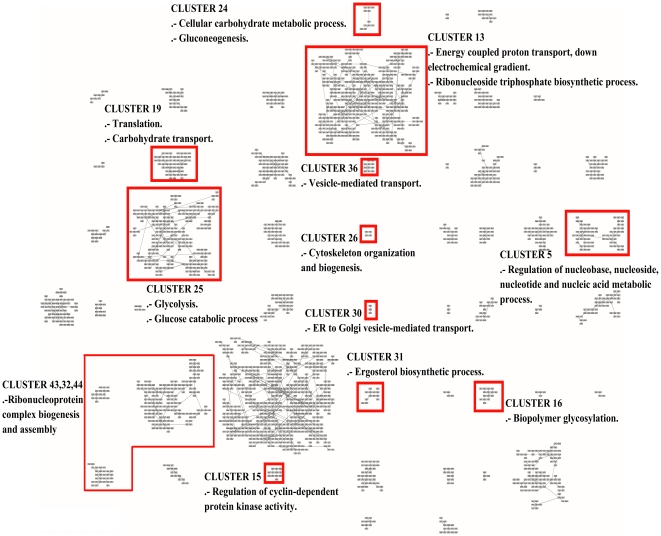
Grid layout of the 2,467 genes probes of Eisen's yeast dataset. As in Figures 13 and 15 we highlight the unique biological annotation associated to each cluster.

**Figure 15:**
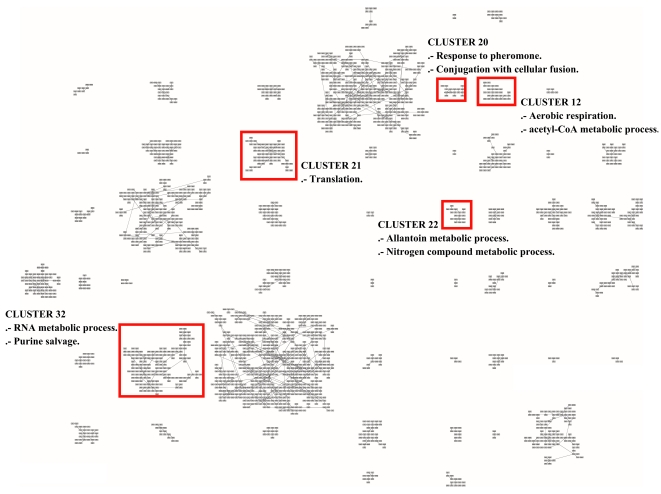
Grid layout of the 2,467 genes probes of Eisen's yeast dataset. As in Figure 13 and 14 we label each cluster with a GO term which has been uniquely and statistically well-associated to a single particular cluster. We refer the reader to the supplementary material (File S4) for an annotation of each cluster.

As a result, from the 52 clusters of genes found by MSTkNN, 31 of them contain significant biological process annotations according to the results obtained with the Gene Ontology Term Finder. The MSTkNN clustering algorithm was able to cluster together (cluster 51) the only four copies of the *asparagine* genes in the data set (ASP3-1, ASP3-2, ASP3-3 and ASP3-4). Those four genes alone have 31 Biological Process GO terms in common. In the supplementary material - File S4 we show the detail of the terms reported for each cluster with a *p-value* 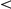
0.01. Additionally in Figure 13, Figure 14 and Figure 15 we show the layout of the clusters of genes with the biological terms found by the Gene Ontology Term Finder with the smaller 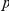-value and that is exclusively present in the cluster. In some of the clusters we also show an extra biological term. For the full list, please refer to the supplementary materials - Files S4 and S5.

There are clusters that share the same main biological process and QAPgrid is able to assign them in contiguous locations in the grid. Clusters 14 and 18 are drawn closely together in Figure 13 because the 12 GO terms with 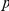-value 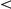
0.01 in cluster 18 are also part of the cluster 14. This is reflecting the capacity of QAPgrid to lay together clusters with similar biological functions. A similar situation can be noted for clusters 39, 35, and 42 also in Figure 13. These three clusters share 8 of the GO terms reported. Additionally, clusters 32, 43 and 44 are the only three clusters that have the GO term “*GO:22613, Ribonucleoprotein complex biogenesis and assembly*” in their biological process annotations and again they were also located in contiguous locations in the grid. These examples show again that the final layout of QAPgrid leaves clusters of genes that share significantly common biological functions in close locations.

## Discussion

In this paper we presented QAPgrid, a methodology for data visualization in a grid layout using a combinatorial optimization problem (The Quadratic Assignment Problem - QAP) as a guide for the assignment. QAPgrid employs a Memetic Algorithm to tackle the large-scale QAP instances created. QAPgrid produces a layout of the objects based on the relationships between them represented by a distance metric. Furthermore, it is also able to incorporate in the layout process the results of a clustering algorithm and a proximity graph. The final layout is produced in two steps: first, the layout of objects in each cluster is computed based on the relationships between objects, then the layout of clusters based on the relationships between the members of the clusters.

We tested QAPgrid using three real world datasets. First, using the distances between Indo European languages to show the usefulness of QAPgrid for data visualization. The layouts obtained by QAPgrid produce a better representation of the global pattern of language similarities when compared with other layout algorithms commonly used, like force-directed algorithms, which tend to get trapped in local optima and do not scale well with the number of elements to visualize/layout. The incorporation of a cluster result allows our approach to co-locate clusters whose members are highly related closer to each other. The QAPgrid was also successfully tested on a large dataset containing information about the ranking of 470 universities. It was able to produce a visualization layout with a high correlation to the ARWU score without the need for “ad hoc” weighting of individual attributes. Finally, we used a microarray dataset for yeast *S*accharomyces cerevisiae that demonstrated the scalability and precision of QAPgrid and its possibility as a novel tool for functional genomics.

In summary, our proposal for data visualization is able to incorporate the result of two other data analysis approaches: clustering and proximity graph modelling. As a result, the final layout represents a synergy of different approaches towards a better representation of the information contained in a dataset. The approach warrants further research in the area to provide scalable parallel computing implementation of QAPgrid to deal with distances matrices between hundreds of thousands of objects in, typically, sets of thousands of different clusters.

## Supporting Information

File S1 Description of the Memetic Algorithm used in this work.
(0.12 MB PDF)

File S2 Clustering of the universities as in Figure 5 (at a higher resolution).
(0.17 MB PDF)

File S3 Members of the clusters for the university ranking data set.
(0.10 MB PDF)

File S4 Gene ontology analysis of genes for the yeast data set.
(0.11 MB PDF)

File S5 Clustering of genes (yeast dataset) as in Figures 13, 14 and 15 (at a higher resolution).
(1.53 MB PDF)
