# Supplementary material for: QAPgrid: A Two Level QAP-Based Approach for Large-Scale Data Analysis and Visualization
Source: PLoS One. 2011 Jan 18;6(1):e14468. doi: 10.1371/journal.pone.0014468 (PMC3022583; doi:10.1371/journal.pone.0014468)
Supplement: File S1 — Description of the Memetic Algorithm used in this work. (0.12 MB PDF) [file pone.0014468.s001.pdf]

The Memetic algorithm for the Quadratic Assignment Problem

The Memetic Algorithm (MA) implemented is presented in Figure 1. In each generation, the algorithm applies the operators: **selectParents**, **recombination**, and two local search strategies (**swapTS** and **8-neighborLS**). Additionally, the algorithm uses an update procedure (**updatePop**), to keep the characteristics of the population structure which is explained later. The MA runs for a certain number of generations ($max\_number\_of\_generations$), which can be either fixed or it can depend on the size of instance and/or the convergence of the algorithm. Extra strategies are applied to deal with premature convergence. Next, we describe each of the components of the MA implemented in more details.

Solution representation

The solution is represented as an array of integers of size n. Each position of the array stores the location of an object, so, $S(i) = k$ means that object i is assigned to location k $(1 \leq i \leq n, 1 \leq k \leq m)$.

Population structure

The population is structured as a ternary hierarchical tree composed of 13 agents as shown in Figure 2. This structure was first employed by Carrizo, Tinetti and Moscato [1] and it has been successfully used in different applications [2–4]. The structure also defines four overlapped subpopulations, each one consisting of a *leader* agent and three *supporter* ones, as shown in Figure 2 using dashed lines. The **updatePop** procedure guarantees that the leader agent of a subpopulation always has the best solution in that subpopulation . As a consequence, the best solution found in the end of each generation will be in $agent^0$ (leader agent of $supop^0$).

Each agent consists of a list of P solutions that are *good quality* and *sufficiently diverse*. In order to ensure these characteristics, the list of solutions in each $agent^i$ is updated as follows. Let $agent^i_j$ represent the solution S_j in the agent i and $d(S_1, S_2)$ the distance between solutions S_1 and S_2 defined by

$$d(S_1, S_2) = |\{i \in \{1, .., n\} \mid S_1(i) \neq S_2(i)\}| \tag{1}$$

First, we say that an agent is *complete* if its list of solutions is full. On the contrary, an agent is not complete. Let $agent^i_{best}$ and $agent^i_{worst}$ represent the solution with the best and worst cost respectively, in the list of solutions of $agent^i$. The **updateAgent**(S, i) in the algorithm in Figure 1, will add the solution S in $agent^i$ if any of the following conditions apply:

1. $agent^i$ is not complete $\wedge$ $d(S, agent^i_k) \geq 0.1n$, $\forall k$.
2. $agent^i$ is not complete $\wedge$ $\exists k / d(S, agent^i_k) < 0.1n$ $\wedge$ $Cost(S) < Cost(agent^i_{best})$. Then S will replace $agent^i_k$ / $d(S, agent^i_k))$ is minimum, $\forall k$.
3. $agent^i$ is complete $\wedge$ $Cost(S) < Cost(agent^i_{best})$. Then S will replace $agent^i_k$ / $d(S, agent^i_k))$ is minimum, $\forall k$.
4. $agent^i$ is complete $\wedge$ $Cost(S) \geq Cost(agent^i_{best}) \wedge d(S, agent^i_k) \geq 0.1n, \forall k \wedge Cost(S) < Cost(agent^i_{worst})$. Then S will replace $agent^i_{worst}$.

When the list is not complete, conditions 1 and 2 decide to add a new solution if it is sufficiently diverse or it is better than the best solution in the list. When the list is complete, conditions 3 and 4 also check if the new solution is better than the best one or it is sufficiently diverse, but there exists an additional condition to decide which solution will be replaced.

Initial population

Each agent in the initial population is initialized with only one solution. Each solution is constructed by assigning each object to a randomly chosen location. Afterwards, the local search **swapTS**, which is described later, is applied. Finally, when we have 13 feasible solutions (one in each agent), the procedure **updatePop** is applied. This process allows us to start our MA with a population composed of local minima solutions.

Parents selection

Our MA restricts the selection of the parents to be recombined based on the tree structure. Figure 3 shows the pseudo-code of the procedure **selectParents**. The parameter i refers to agents 1 to 12 and the variable k represents the leader agent of the agent i in the tree structure. For example, if $i = 1$, 2 or 3, then $k = 0$; if $i = 4$, 5 or 6, then $k = 1$, and so on. For each i, the $parent_1$ is a solution selected uniformly at random from the pool of solutions of $agent^i$. The selection of $parent_2$ will depend on the diversity of the $subpop^k$. We say that a subpopulation is diverse (heterogenous), if the supporters of the subpopulation have less than 20% of the objects on the same location, else it will be declared to be "no diverse" (homogeneous). In the first case, $parent_2$ is chosen uniformly at random from the pool of solutions of $agent^k$. On the contrary, if the subpopulation is homogeneous, $parent_2$ is randomly chosen from an $agent^j$, such that the subpopulation of $agent^j$ is not k. For example, if $i = 4$ and $subpop^1$ ($k = 1$) is homogeneous, then $parent_2$ will be randomly chosen from $agent^j$, such that $j \in \{7, 8, 9, 10, 11, 12\}$, since $\{4, 5, 6\}$ belong to $subpop^1$. On the contrary, if $subpop^1$ ($k = 1$) has **not** lost diversity, then $parent_2$ will be chosen randomly from the $agent^1$.

Recombination operator

We use a modified version of the *cycle crossover* also used by Merz and Freisleben [5]. The operator aims to produce an offspring with no extra mutation from the parents (an information-preserving crossover), which means that each position in the offspring comes from one of the parents. The original cycle crossover was designed to be used in QAP instances with $n = m$. When we use it on an instance with $n < m$, an object can be left without a location. To explain the recombination operator, we use the example shown in Figure 4.

Initially, all the objects with the same location in both parents are copied to the offspring (objects **A** and **E**). Then, the algorithm randomly selects an unassigned object from the offspring, say object **D**, and looks at its location in one of the parents, say Parent 2. Thus, the recombination assigns location #3 to element **D**. Next, we look at the location of **D** in Parent 1 (i.e. location #1) and check which object is in location #1 in Parent 2 (i.e. object **G**), assigning its location to the offspring (i.e. object **G** goes to location #1). The process is repeated, now checking the location of object **G** in Parent 1 (location #4). However, as location #4 is not present in Parent 2, the process stops. We repeat the process starting with object **H** in parent 1. After processing all the objects in the offspring, object **B** still does not have a location because both locations #3 and #12 have already been taken. This situation does not happen when $n = m$. To deal with this problem, for each of the unassigned objects we trace a straight line between the location of object **B** in both parents (locations #3 and #12) and choose a random location over it, in this case location #6. It can also be the case that all the locations in that line are already taken. In that situation the algorithm randomly selects an unassigned location from any of the parents.

Tabu Search

Tabu Search (TS) [6] is a metaheuristic that uses memory structures to avoid a local search strategy to be trapped in a local minima. The inclusion of Tabu Search in the local search strategy in the

Memetic Algorithm (first proposed by Moscato [7]) has consistently shown very good results in different applications [2, 7, 8] and its has been chosen due to the proved synergy within this population-based appraoch.

We use a basic TS algorithm to enhance the *pairwise interchange heuristic* (**swapTS** in the MA algorithm of Figure 1). In the pairwise interchange heuristic, the neighborhood of a solution, $N(S)$, corresponds to the set of all new solutions produced by the swap of the locations of two different objects, i.e, $S(i) = k$ is swapped with $S(i') = k'$, producing $S(i) = k'$ and $S(i') = k$. In each iteration, the best solution from the neighborhood $N(S)$ is selected. For the TS version, after swap $S(i) = k$ with $S(i') = k'$, the objects i and i' are forbidden to return to the locations k and k', respectively, for a certain number of iterations (*tabu tenure*). However, if the swap improves the value of the objective function of the best solution found so far, then it is allowed (*aspiration criteria*). The process is repeated until there is no improvement for a fixed number of iterations.

The tabu tenure is an integer value that is randomly generated from an interval $[T_1, T_2]$ after each swap is performed.

8-neighbor local search

The second local search that the MA uses is the greedy algorithm called **8-neighborLS**. This local search aims at exploring the repository of objects to contiguous locations in the grid. Due to the representation used, a solution only considers the locations that are already assigned, so the operators do not allow the algorithm to fully explore all locations of the grid.

This local search is applied once per generation on the best solution of the population and only if $n < m$ (the number of objects is smaller than the number of locations). On the contrary, if $n = m$, there is no need to use it, since we are already using the whole set of grid locations available.

The algorithm works as follows: for each object i, the algorithm tests if moving the selected object to one of its 8 contiguous locations in the grid improves the quality of the solution. In case it does, the object is moved and the process is repeated again, until no further improvement can be obtained moving that object. The same process is repeated with each object.

Diversification

One of the problems that a designer of a population-based metaheuristic needs to address is the premature convergence of the population [7]. This situation generally happens when the size of the population is small, like in this case. In order to avoid this, two diversification strategies have been implemented. The first one was already described previously, which aims to avoid convergence in a subpopulation by selecting parents from different subpopulations to perform the recombination operator.

The second strategy is a mechanism that is triggered when the MA has been unable to find a better solution during the last $n/4$ generations. If that is the case, the whole population is restarted and only the best solution from $agent^0$ is kept. This strategy aims to provide a new starting point for the MA, without losing the best individual found so far.

Some preliminary results

The MA was coded in Java 1.6 and the computational tests were run on a PC with Intel Core 2 Duo CPU (1.86 Ghz, 2GB RAM) running Solaris 10. We analyzed the performance of the MA using the instances from the Quadratic Assignment Problem Library (QAPLIB) [9]. QAPLIB is a repository of instances of the Quadratic Assignment Problem that can be used as a benchmark for new algorithms. The instances considered have the same number of objects (n) and number of locations (m), and range from 25 to 256 objects.

We compared our MA with two other population-based metaheuristics. The first one was presented by Demirel and Toksari [10]. It uses an Ant Colony System that implements a Simulated Annealing local search (ACSA). The second one corresponds to a MA proposed by Merz and Freisleben [11] (MA_M). The algorithm uses the information-preserving crossover and a variant of the pairwise interchange heuristic on an unstructured population of 40 individuals. Complementary, Merz and Freisleben in [5], compare the MA against five competitors. We used the results from the later, since the results are better than [11]. In both algorithms (ACSA and MA_M), the authors also use the instances taken from the QAPLIB [9].

In general, the MA proposed performed well compared with both competitors. In the case of ACSA, our algorithm outperformed in all but two instances (*tai50a* and *tai80a*). The average gap is 0.115%, compared to 0.382% of ACSA. In comparison with MA_M we also obtained a slight better average gap (0.322% against 0.388%), but the difference is not so significant.

References

1. Carrizo J, Tinetti F, Moscato P (1992) A computational ecology for the quadratic assignment problem. In: Proceedings of the 21st Meeting on Informatics and OR. Buenos Aires, Argentina, August.

2. Berretta R, Moscato P (1999) The number partitioning problem: An open challenge for evolutionary computation? In: Corne D, Dorigo M, editors, New Ideas in Optimization, McGraw-Hill. pp. 261–278.

3. Berretta R, Rodrigues L (2004) A memetic algorithm for a multistage capacitated lot-sizing problem. Int J of Production Economics 87: 67–81.

4. Buriol L, Franca P, Moscato P (2004) A new memetic algorithm for the asymmetric traveling salesman problem. J of Heuristics 10: 483–506.

5. Merz P, Freisleben B (2000) Fitness landscape analysis and memetic algorithms for the quadratic assignment problem. IEEE Transactions on Evol Computation 4: 337–352.

6. Glover F, Laguna M (1997) Tabu Search. Norwell, Massachusetts: Kluwer Academic Publishers.

7. Moscato P (1993) An introduction to population approaches for optimization and hierarchical objective functions: A discussion on the role of tabu search. Annals of OR 41: 85–121.

8. Moscato P, Mendes A, Berretta R (2007) Benchmarking a memetic algorithm for ordering microarray data. Biosystems 88: 56–75.

9. Burkard R, Karisch S, Rendl F (1997) QAPLIB - a quadratic assignment problem library. J of Global Optimization 10: 391–403.

10. Demirel N, Toksari M (2006) Optimization of the quadratic assignment problem using an ant colony algorithm. Applied Mathematics and Computation 183: 427—435.

11. Merz P, Freisleben B (1999) A comparison of memetic algorithm, tabu search, and ant colonies for the quadratic assignment problem. In: Angeline P, editor, Congress on Evol. Computation (CEC'99). IEEE Press, pp. 2063–2070.

```
MA()
    pop = initializePop()
    REPEAT
        FOR i=1 TO 12
            selectParents(i,parent_1,parent_2)
            offspring = recombination(parent_1,parent_2)
            swapTS(offspring)
            updateAgent(offspring,i)
        END FOR
        updatePop(pop)
        IF (m > n) /*more locations than objects*/
            8-neighborLS(pop)
        END IF
        IF popConverged(pop)
            pop = restartPop(pop)
        END IF
    UNTIL max_number_of_generations
END
```

Figure 1. Pseudo-code of the Memetic Algorithm implemented for the Quadratic Assignment Problem.

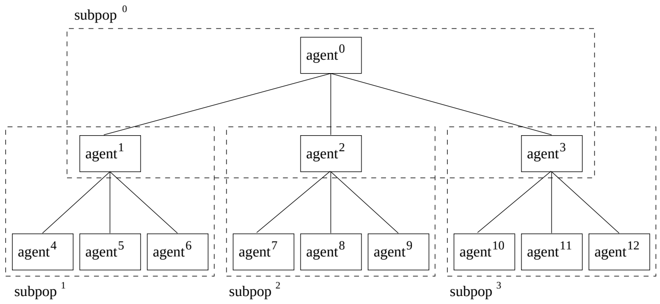

Figure 2. Population structure used in our memetic algorithm. It has been shown before that the use of population structures is a useful mechanism to bias the search process, and that accelerates the discovery of near-optimal solutions. We have used a hierarchical population composed of 13 agents which are organized in a complete ternary tree. The figure also indicates the subpopulations present in the structure.

```
selectParents(i, parent_1, parent_2)
      k = (i − 1)/3
      IF (lostDiversity(subpop^k))
            j = U[4,12]/ agent^j ∉ subpop^k
      ELSE
            j = (i − 1)/3
      END IF
      parent_1 = randomIndividual(agent^i)
      parent_2 = randomIndividual(agent^j)
END
```

Figure 3. Pseudo-code of the selectionParents procedure from the Memetic Algorithm showed in Figure 1.

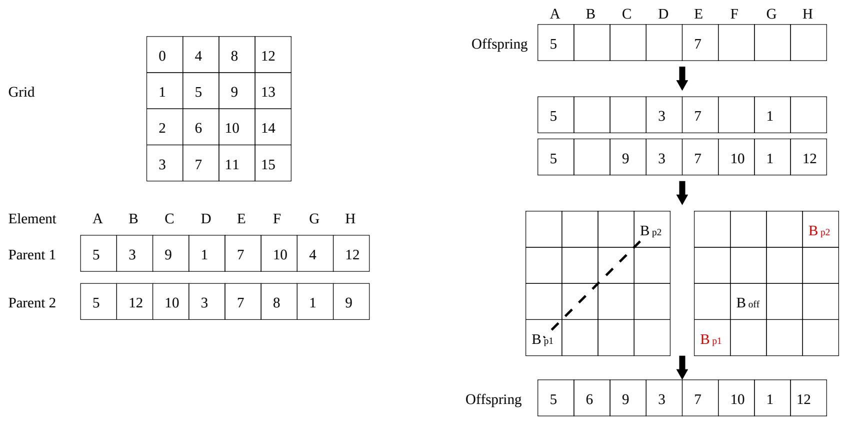

Figure 4. Example of the modified cycle crossover operator for the memetic algorithm. It solves the problem of unassigned objects of the original cycle crossover in the case when $n < m$.
