# Supplementary figures and images for: QAPgrid: A Two Level QAP-Based Approach for Large-Scale Data Analysis and Visualization
Source: PLoS One. 2011 Jan 18;6(1):e14468. doi: 10.1371/journal.pone.0014468 (PMC3022583; doi:10.1371/journal.pone.0014468)

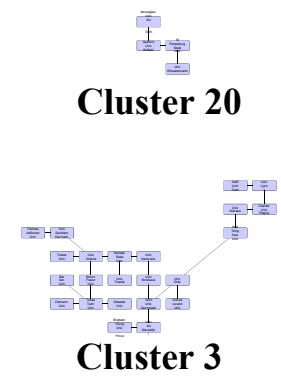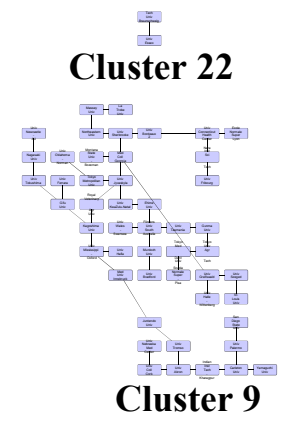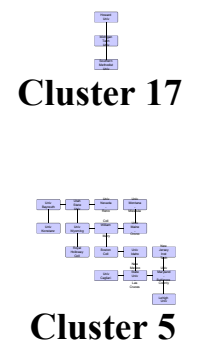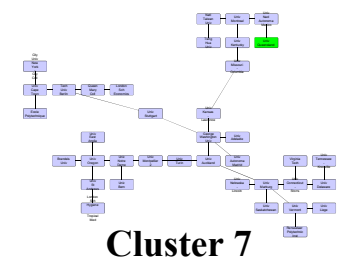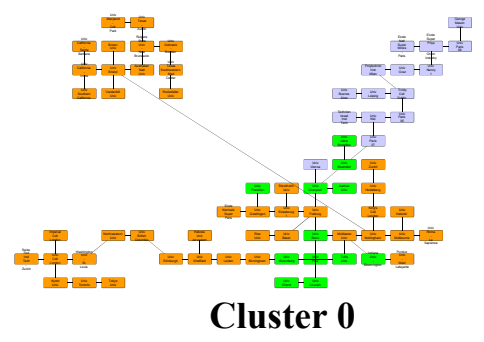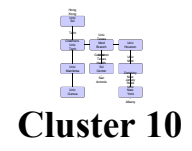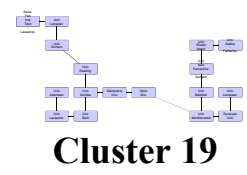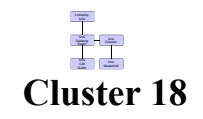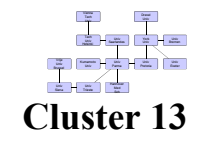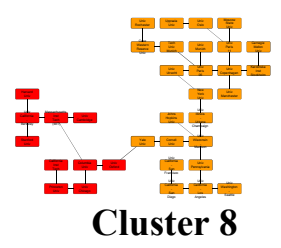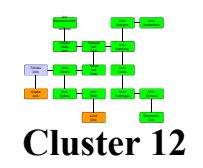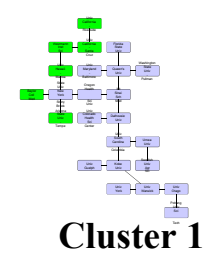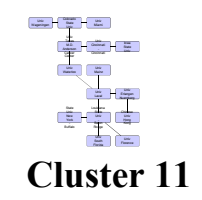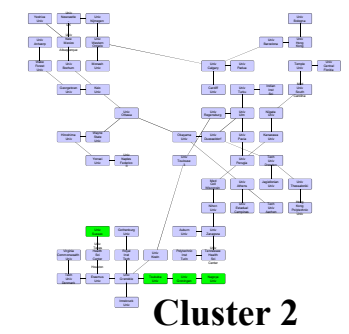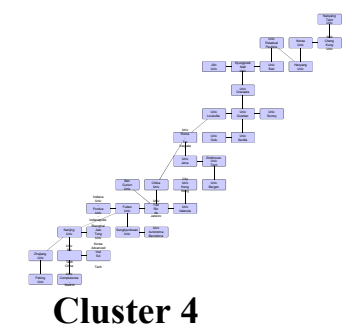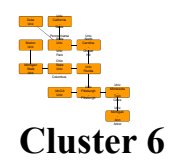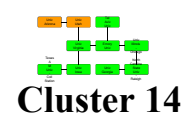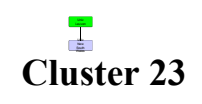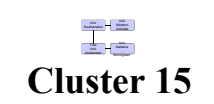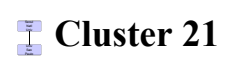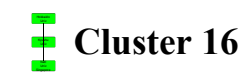

Supplement: File S2 — Clustering of the universities as in Figure 5 (at a higher resolution). (0.17 MB PDF) [file pone.0014468.s002.pdf]
