# Supplementary material for: QAPgrid: A Two Level QAP-Based Approach for Large-Scale Data Analysis and Visualization
Source: PLoS One. 2011 Jan 18;6(1):e14468. doi: 10.1371/journal.pone.0014468 (PMC3022583; doi:10.1371/journal.pone.0014468)
Supplement: File S3 — Members of the clusters for the university ranking data set. (0.10 MB PDF) [file pone.0014468.s003.pdf]

Members of the clusters for the university ranking data set.

Cluster 0 (70): Aarhus U, Australian Natl U, Brown U, Ecole Natl Super Mines Paris Ecole Normale Super Paris, Ecole Super Phys Chem Industry, George Mason U, Hebrew U Jerusalem, Imperial Coll London, Indiana U - Bloomington, King's Coll London, Kyoto U, McMaster U, Northwestern U, Pol Inst Milan, Purdue U West Lafayette, Rice U, Rockefeller U, Rutgers State U New Brunswick, Stockholm U, Swiss Fed Inst Tech, Technion Israel I T, Tokyo U, Trinity Coll Dublin, Tufts U, U Basel, U Birmingham, U Bonn, U Bristol, U British Columbia, U Buenos Aires, U California Irvine, U California Santa Barbara, U Coll London,U Colorado Boulder, U Edinburgh, U Frankfurt, U Freiburg, U Ghent, U Goettingen, U Graz, U Heidelberg, U Helsinki, U Kiel, U Leiden, U Leipzig, U Libre Bruxelles, U Liverpool, U Louvain, U Maryland Coll Park, U Melbourne, U Muenster, U Nancy 1, U Nottingham, U Paris 05, U Paris 07, U Paris 09, U Pisa, U Roma La Sapienza, U Sheffield, U Southern California, U Strasbourg 1, U Texas Austin, U Texas Southwestern Med Center, U Toronto, U Vienna, U Wuerzburg, U Zurich, Vanderbilt U, Washington U St. Louis

Cluster 1 (24): Arizona State U Tempe, Baylor Coll Med, Dalhousie U, Florida State U, Kobe U, Mt Sinai Sch Med, Oregon Health Sci U, Pohang U Sci Tech, Queen's U, State U New York Stony Brook, Swedish U Agr Sci, Umea U, U California Riverside, U California Santa Cruz, U Colorado Health Sci Center, U Guelph, U Hawaii Manoa, U Maryland Baltimore, U Otago, U South Carolina Columbia, U Warwick, U York, Washington State U Pullman, Weizmann I Sci

Cluster 2 (62): Auburn U, Cardiff U, Erasmus U, Georgetown U, Gothenburg U, Hiroshima U, Hong Kong Polytechnic U, Indian Inst Sci, Innsbruck U, Jagiellonian U, Kanazawa U, Keio U, Med Coll Wisconsin, Med U South Carolina, Monash U,Nagoya U, Nihon U, Niigata U, Okayama U, Polytechnic Inst Turin, Royal I T, Tech U Aachen, Tech U Denmark, Tech U Dresden, Temple U, Tsukuba U, U Antwerp, U Athens, U Barcelona, U Bochum, U Bologna, U Calgary, U CentralFlorida, U Duesseldorf, U Estadual Campinas, U Grenoble 1, U Groningen, U HongKong, U Koeln, U Naples Federico II, U New Mexico Albuquerque, U Newcastle UK, U Nijmegen, U Ottawa, U Padua, U Pavia, U Perugia, U Regensburg, U Sussex, U Tennessee Health Sci Center, U Texas Health Sci Center Houston, U Thessaloniki, U Toulouse 3, U Turku, U Ulm, U Western Ontario, U Zaragoza, VirginiaCommonwealth U, Wake Forest U, Wayne State U, Yeshiva U, Yonsei U

Cluster 3 (23): Bar Ilan U, Brigham Young U - Provo, Charles U Prague, Clemson U, Delft U Tech, Eotvos Lorand U, Kansas State U, Natl Tsing Hua U, Simon Fraser U, Tech UDarmstadt, Texas Tech U, Thomas Jefferson U, Tulane U, U Aix Marseille 1,U Bordeaux 1, U Chile, U Karlsruhe, U Lyon 1, U Southern Denmark, U Twente, U Victoria, U Warsaw, Waseda U

Cluster 4 (34): Ben Gurion U, Chiba U, City U Hong Kong, Eindhoven U Tech, Fudan U, Indiana U-Purdue U, Jilin U, Korea Advanced Inst Sci Tech, Kyungpook Natl U, Nanjing U, Nanyang Tech U, Natl Cheng Kung U, Peking U, Shangai Jiao Tong U, Sungkyunkwan U, U Autonoma Barcelona, U Bari, U Bergen, U Complutense Madrid, U Estadual Paulista, U Fed Rio de Janeiro, U Granada, U Jena, U Louisville, U Oulu, U Quebec, U Roma Tor Vergata, U Sci Tech China, U Sevilla, U Surrey, U Valencia, Zhejiang U, Hanyang U, Korea U

Cluster 5 (16): Boston Coll, Coll William Mary, Lehigh U, New Jersey Inst Tech, New Mexico State U Las Cruces, Royal Holloway Coll, U Bayreuth, U Cagliari, U Idaho, U Konstanz, U Maine Orono, U Maryland Baltimore County, U Montana Missoula, U Nevada Reno, U Wyoming, Utah State U

Cluster 6 (12): Boston U, Duke U, McGill U, Michigan State U, Ohio State U Columbus, Pennsylvania State U, U Park, U California Davis, U Florida, U MichiganAnn Arbor, U Minnesota Twin Cities, U North Carolina Chapel Hill, U Pittsburgh

Cluster 7 (38): Brandeis U, City U New York City Coll, Ecole Polytechnique, George Washington U, London Sch Economics, London Sch Hygiene Tropical Med, Natl Taiwan U, Queen Mary Coll, Rensselaer Polytechnic Inst, Tech U Berlin,Tsing Hua U, U Adelaide, U Auckland, U Autonoma Madrid, U Kansas Lawrence,U Bern, U Cape Town, U Connecticut Storrs, U Delaware, U East Anglia, U Kentucky, U Liege, U Marburg, U Missouri Columbia, U Montpellier 2,U Montreal, U Nacl Autonoma Mexico, U Nebraska Lincoln, U Notre Dame,U Oregon, U Queensland, U Saskatchewan, U St Andrews, U Stuttgart, U Tennessee Knoxville, U Turin, U Vermont, Virginia Tech \hline

Cluster 8 (35): California Inst Tech, Carnegie Mellon U, Case Western Reserve U, Columbia U, Cornell U, Harvard U, Johns Hopkins U, KarolinskaInst Stockholm, Massachusetts Inst Tech, Moscow State U, New York U, Princeton U, Stanford U, Tech U Munich, U California Berkeley,U California Los Angeles, U California San Diego, U California SanFrancisco, U Cambridge, U Chicago, U Copenhagen, U Illinois Urbana Champaign, U Manchester, U Munich, U Oslo, U Oxford, U Paris 06,U Paris 11, U Pennsylvania, U Rochester, U Utrecht, U WashingtonSeattle, U Wisconsin Madison, Uppsala U, Yale U

Cluster 9 (49): Carleton U, Ecole Normale Super Lyon, Ehime U, Flinders U South Australia, Gifu U, Gunma U, Indian Inst Tech Kharagpur, Juntendo U, Kagoshima U, La Trobe U, Massey U, Med Coll Georgia, Med U Innsbruck, U Bradford, Murdoch U, Nagasaki U, Nara Inst Sci Tech, Northeastern U, Royal Veterinary Agr U, San Diego State U, Scuola Normale Super Pisa, St. Louis U, Tokyo Med Dent U, Tokyo Metropolitan U, Tokyo U Agr Tech, U Akron, U Bordeaux 2, U Coll Cork, U Connecticut Health Center, U Ferrara, U Fribourg, U Greifswald, U Haifa, U Halle Wittenberg, U Jyvaskyla, U KwaZulu Natal, U Mississippi Oxford, Med Center, \textbf{U Newcastle AU}, U Oklahoma Norman, U Palermo, U Sherbrooke, U Szeged, U Nebraska, U Tasmania, U Tokushima, U Tromso, U Wales Swansea, Yamaguchi U., Montana State U Bozeman

Cluster 10 (9): Chalmers U Tech, Hong Kong U Sci Tech, State U New York Albany, U Genoa,U Houston, U Manitoba, U Med Dentistry New Jersey, U Texas Health Sci Center San Antonio, U Texas Med Branch Galveston

Cluster 11 (15): Chinese U Hong Kong, Colorado State U, Iowa State U, Louisiana State U BatonRouge, State U New York Buffalo, U Cincinnati Cincinnati, U Erlangen Nuernberg,U Florence, U Laval, U Mainz, U Miami, U South Florida, U Texas M.D. AndersonCancer Center, U Wageningen, U Waterloo

Cluster 12 (17): Dartmouth Coll, Georgia Inst Tech, Lund U, Oregon State U, Osaka U, Tohoku U,Tokyo Inst Tech, U Alberta, U Amsterdam, U Geneva, U Glasgow, U Hamburg,U Leeds, U Massachusetts Amherst, U Milan, U Sydney, U Tuebingen

Cluster 13 (14): Drexel U, Hannover Med Sch, Kumamoto U, Tech U Helsinki, U Bremen, U Exeter,U Pretoria, U Saarlandes, U Siena, U Trieste, Vienna Tech U, Vrije U Brussel,U Parma, York U

Cluster 14 (10): Emory U, North Carolina State U Raleigh, Tel Aviv U, Texas AMU Coll Station,U Arizona, U Georgia, U Illinois Chicago, U Iowa, U Utah, U Virginia

Cluster 15 (4): Free U Amsterdam, U Alabama Birmingham, U Southampton, U Western Australia

Cluster 16 (3): Hokkaido U, Kyushu U, Natl U Singapore

Cluster 17 (3): Howard U, Michigan Tech U, Southern Methodist U

Cluster 18 (5): Linkoping U, U Coll Dublin, U Duisburg Essen, U Giessen, U Maastricht

Cluster 19 (17): Macquarie U, Open U, Swiss Fed Inst Tech - Lausanne, Syracuse U, U Aberdeen,U Alaska Fairbanks, U Bath, U Bielefeld, U Dundee, U Durham, U Lancaster,U Lausanne, U Leicester, U Mediterranee, U New Hampshire Durham, U Reading,U Rhode Island

Cluster 20 (4): Norwegian U Sci Tech, Queen's U Belfast, St Petersburg State U, U Witwatersrand

Cluster 21 (2): Seoul Natl U, U Sao Paulo

Cluster 22 (2): Tech U Braunschweig, U Essex

Cluster 23 (2): U Leuven, U New South Wales
