# Supplementary material for: QAPgrid: A Two Level QAP-Based Approach for Large-Scale Data Analysis and Visualization
Source: PLoS One. 2011 Jan 18;6(1):e14468. doi: 10.1371/journal.pone.0014468 (PMC3022583; doi:10.1371/journal.pone.0014468)
Supplement: File S4 — Gene ontology analysis of genes for the yeast data set. (0.11 MB PDF) [file pone.0014468.s004.pdf]

Gene Ontology analysis of genes for yeast data set

This file contains the details of the Gene Ontology Analysis performed on the genes of the yeast data set. For 31 out of 52 clusters the Gene Ontology Term Finder website[1] was able to find common biological process for the set of genes of the cluster. We consider only those terms with a significance *P-value* $<$ 0.01. In order to help with the reading of the results, in Figure 1 we show the grid layout of the clusters resulting from the application of **QAPgrid** and the id of the cluster in each cell of the grid. Clusters in yellow did not report any common biological process.

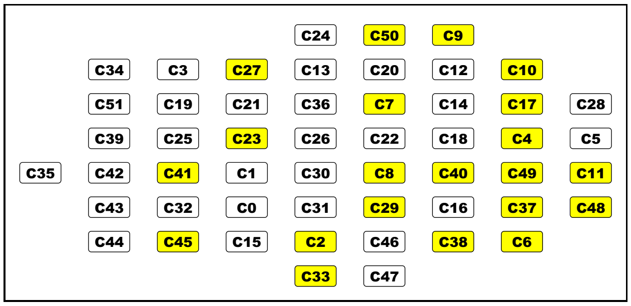

Figure 1: Grid layout of the clusters of genes of yeast. Cells in yellow do not have any common biological process reported by Gene Ontology Term Finder.

Table 1 shows the Gene Ontology terms related with the common biological process in which genes of a cluster are involved. The first column indicates the number of clusters that contain that term. For example the term *cell cycle process* in cluster 0 has a number two, which means that this term is present in two clusters (cluster 0 and cluster 47). The second and the third columns indicate the code and the name of the GO term, respectively. Columns **Cluster** and **Background** reflect how many genes are annotated to that GO term in the Cluster and in the total Background (the background used is the whole set of 2,467 genes). The last column shows the *P-value* of the term, we only searched for terms with a *P-value* $<$ 0.01. For details on how the *P-value* is calculated, we refer to the website of the application.

[1] *http://db.yeastgenome.org/cgi-bin/GO/goTermFinder.pl*

Table 1: Biological process of clusters of genes for the yeast data set according to the information provided by Gene Ontology Term Finder.

Rep	GOID	GO-term	Frequency		*P-value*
			Cluster	Background	
	**Cluster**	0			
1	279	M phase	52/592 genes, 8.8%	105/2464 genes, 4.3%	4.84E-06
1	7049	cell cycle	95/592 genes, 16.0%	241/2464 genes, 9.8%	1.24E-05
6	9059	macromolecule biosynthetic process	137/592 genes, 23.1%	391/2464 genes, 15.9%	4.39E-05
2	22402	cell cycle process	83/592 genes, 14.0%	210/2464 genes, 8.5%	0.0001
1	70	mitotic sister chromatid segregation	18/592 genes, 3.0%	24/2464 genes, 1.0%	0.00014
1	819	sister chromatid segregation	18/592 genes, 3.0%	24/2464 genes, 1.0%	0.00014
1	7059	chromosome segregation	25/592 genes, 4.2%	40/2464 genes, 1.6%	0.00017
1	6259	DNA metabolic process	89/592 genes, 15.0%	232/2464 genes, 9.4%	0.00018
1	7017	microtubule-based process	30/592 genes, 5.1%	54/2464 genes, 2.2%	0.0004
1	51276	chromosome organization and biogenesis	115/592 genes, 19.4%	327/2464 genes, 13.3%	0.00058
1	6310	DNA recombination	40/592 genes, 6.8%	83/2464 genes, 3.4%	0.00077
2	6260	DNA replication	44/592 genes, 7.4%	96/2464 genes, 3.9%	0.00126
1	51321	meiotic cell cycle	31/592 genes, 5.2%	59/2464 genes, 2.4%	0.00132
1	51327	M phase of meiotic cell cycle	31/592 genes, 5.2%	59/2464 genes, 2.4%	0.00132
1	7126	meiosis	31/592 genes, 5.2%	59/2464 genes, 2.4%	0.00132
6	6412	translation	102/592 genes, 17.2%	293/2464 genes, 11.9%	0.0049
1	22403	cell cycle phase	67/592 genes, 11.3%	176/2464 genes, 7.1%	0.00923
	**Cluster**	1			
1	19725	cellular homeostasis	9/37 genes, 24.3%	67/2464 genes, 2.7%	4.44E-05
1	42592	homeostatic process	9/37 genes, 24.3%	69/2464 genes, 2.8%	5.77E-05
1	55082	cellular chemical homeostasis	8/37 genes, 21.6%	60/2464 genes, 2.4%	0.00024
1	6873	cellular ion homeostasis	8/37 genes, 21.6%	60/2464 genes, 2.4%	0.00024
1	48878	chemical homeostasis	8/37 genes, 21.6%	62/2464 genes, 2.5%	0.00031
1	50801	ion homeostasis	8/37 genes, 21.6%	62/2464 genes, 2.5%	0.00031
1	30641	regulation of cellular pH	5/37 genes, 13.5%	16/2464 genes, 0.6%	0.00031
1	45851	pH reduction	5/37 genes, 13.5%	16/2464 genes, 0.6%	0.00031
1	51452	intracellular pH reduction	5/37 genes, 13.5%	16/2464 genes, 0.6%	0.00031
1	51453	regulation of intracellular pH	5/37 genes, 13.5%	16/2464 genes, 0.6%	0.00031
1	7035	vacuolar acidification	5/37 genes, 13.5%	16/2464 genes, 0.6%	0.00031
1	6885	regulation of pH	5/37 genes, 13.5%	18/2464 genes, 0.7%	0.0006
1	30003	cellular cation homeostasis	7/37 genes, 18.9%	55/2464 genes, 2.2%	0.0016
1	30004	cellular monovalent inorganic cation homeostasis	5/37 genes, 13.5%	22/2464 genes, 0.9%	0.00176
1	55080	cation homeostasis	7/37 genes, 18.9%	57/2464 genes, 2.3%	0.00203
2	45047	protein targeting to ER	5/37 genes, 13.5%	24/2464 genes, 1.0%	0.00278
1	55067	monovalent inorganic cation homeostasis	5/37 genes, 13.5%	24/2464 genes, 1.0%	0.00278
1	7033	vacuole organization and biogenesis	6/37 genes, 16.2%	41/2464 genes, 1.7%	0.00321
	**Cluster**	3			
1	15749	monosaccharide transport	5/26 genes, 19.2%	22/2464 genes, 0.9%	0.00025
1	8645	hexose transport	5/26 genes, 19.2%	22/2464 genes, 0.9%	0.00025
2	8643	carbohydrate transport	5/26 genes, 19.2%	25/2464 genes, 1.0%	0.00049
	**Cluster**	5			
1	19219	regulation of nucleobase, nucleoside, nucleotide and nucleic acid metabolic process	21/69 genes, 30.4%	295/2464 genes, 12.0%	0.00728
	**Cluster**	12			
2	6091	generation of precursor metabolites and energy	17/29 genes, 58.6%	107/2464 genes, 4.3%	4.62E-15
1	9060	aerobic respiration	14/29 genes, 48.3%	57/2464 genes, 2.3%	9.83E-15
1	45333	cellular respiration	14/29 genes, 48.3%	58/2464 genes, 2.4%	1.28E-14
1	51187	cofactor catabolic process	9/29 genes, 31.0%	16/2464 genes, 0.6%	8.49E-13
1	9109	coenzyme catabolic process	9/29 genes, 31.0%	16/2464 genes, 0.6%	8.49E-13
1	15980	energy derivation by oxidation of organic compounds	14/29 genes, 48.3%	83/2464 genes, 3.4%	2.90E-12
					It continues on next page

Table 1 – continued from previous page

Rep	GOID	GO-term	Frequency		*P-value*
			Cluster	Background	
1	6084	acetyl-CoA metabolic process	9/29 genes, 31.0%	20/2464 genes, 0.8%	1.21E-11
1	46356	acetyl-CoA catabolic process	8/29 genes, 27.6%	15/2464 genes, 0.6%	5.57E-11
1	6099	tricarboxylic acid cycle	8/29 genes, 27.6%	15/2464 genes, 0.6%	5.57E-11
1	6118	electron transport	9/29 genes, 31.0%	23/2464 genes, 0.9%	5.76E-11
1	42773	ATP synthesis coupled electron transport	8/29 genes, 27.6%	18/2464 genes, 0.7%	3.70E-10
1	42775	organelle ATP synthesis coupled electron transport	8/29 genes, 27.6%	18/2464 genes, 0.7%	3.70E-10
1	51186	cofactor metabolic process	12/29 genes, 41.4%	91/2464 genes, 3.7%	6.83E-09
1	6732	coenzyme metabolic process	11/29 genes, 37.9%	70/2464 genes, 2.8%	7.14E-09
2	6119	oxidative phosphorylation	8/29 genes, 27.6%	30/2464 genes, 1.2%	4.52E-08
1	6121	mitochondrial electron transport, succinate to ubiquinone	4/29 genes, 13.8%	4/2464 genes, 0.2%	1.10E-06
1	16310	phosphorylation	8/29 genes, 27.6%	74/2464 genes, 3.0%	8.29E-05
1	6793	phosphorus metabolic process	8/29 genes, 27.6%	101/2464 genes, 4.1%	0.0009
1	6796	phosphate metabolic process	8/29 genes, 27.6%	101/2464 genes, 4.1%	0.0009
1	6100	tricarboxylic acid cycle intermediate metabolic process	4/29 genes, 13.8%	18/2464 genes, 0.7%	0.003
1	6122	mitochondrial electron transport, ubiquinol to cytochrome c	3/29 genes, 10.3%	7/2464 genes, 0.3%	0.00353
	**Cluster**	13			
2	6119	oxidative phosphorylation	22/427 genes, 5.2%	30/2464 genes, 1.2%	1.14E-08
1	15985	energy coupled proton transport, down electrochemical gradient	12/427 genes, 2.8%	12/2464 genes, 0.5%	4.51E-07
1	15986	ATP synthesis coupled proton transport	12/427 genes, 2.8%	12/2464 genes, 0.5%	4.51E-07
1	46034	ATP metabolic process	12/427 genes, 2.8%	12/2464 genes, 0.5%	4.51E-07
1	6754	ATP biosynthetic process	12/427 genes, 2.8%	12/2464 genes, 0.5%	4.51E-07
1	9144	purine nucleoside triphosphate metabolic process	12/427 genes, 2.8%	13/2464 genes, 0.5%	4.95E-06
1	9145	purine nucleoside triphosphate biosynthetic process	12/427 genes, 2.8%	13/2464 genes, 0.5%	4.95E-06
1	9205	purine ribonucleoside triphosphate metabolic process	12/427 genes, 2.8%	13/2464 genes, 0.5%	4.95E-06
1	9206	purine ribonucleoside triphosphate biosynthetic process	12/427 genes, 2.8%	13/2464 genes, 0.5%	4.95E-06
1	9199	ribonucleoside triphosphate metabolic process	13/427 genes, 3.0%	15/2464 genes, 0.6%	5.69E-06
1	9201	ribonucleoside triphosphate biosynthetic process	13/427 genes, 3.0%	15/2464 genes, 0.6%	5.69E-06
1	9142	nucleoside triphosphate biosynthetic process	14/427 genes, 3.3%	18/2464 genes, 0.7%	1.98E-05
1	15992	proton transport	12/427 genes, 2.8%	15/2464 genes, 0.6%	0.00012
1	6818	hydrogen transport	12/427 genes, 2.8%	15/2464 genes, 0.6%	0.00012
1	9141	nucleoside triphosphate metabolic process	14/427 genes, 3.3%	20/2464 genes, 0.8%	0.00018
2	6091	generation of precursor metabolites and energy	39/427 genes, 9.1%	107/2464 genes, 4.3%	0.00067
1	6970	response to osmotic stress	20/427 genes, 4.7%	39/2464 genes, 1.6%	0.00074
1	15672	monovalent inorganic cation transport	12/427 genes, 2.8%	19/2464 genes, 0.8%	0.00696
1	9628	response to abiotic stimulus	23/427 genes, 5.4%	55/2464 genes, 2.2%	0.00967
	**Cluster**	14			
2	6508	proteolysis	25/48 genes, 52.1%	107/2464 genes, 4.3%	6.08E-21
2	19941	modification-dependent protein catabolic process	23/48 genes, 47.9%	93/2464 genes, 3.8%	1.43E-19
2	51603	proteolysis involved in cellular protein catabolic process	23/48 genes, 47.9%	93/2464 genes, 3.8%	1.43E-19
2	6511	ubiquitin-dependent protein catabolic process	23/48 genes, 47.9%	93/2464 genes, 3.8%	1.43E-19
2	43632	modification-dependent macromolecule catabolic process	23/48 genes, 47.9%	96/2464 genes, 3.9%	3.20E-19
2	44257	cellular protein catabolic process	23/48 genes, 47.9%	98/2464 genes, 4.0%	5.38E-19
2	30163	protein catabolic process	23/48 genes, 47.9%	104/2464 genes, 4.2%	2.36E-18
2	43285	biopolymer catabolic process	25/48 genes, 52.1%	168/2464 genes, 6.8%	8.65E-16
2	44265	cellular macromolecule catabolic process	25/48 genes, 52.1%	182/2464 genes, 7.4%	6.50E-15
2	9057	macromolecule catabolic process	25/48 genes, 52.1%	203/2464 genes, 8.2%	9.79E-14
3	44248	cellular catabolic process	25/48 genes, 52.1%	258/2464 genes, 10.5%	3.20E-11
3	9056	catabolic process	25/48 genes, 52.1%	264/2464 genes, 10.7%	5.51E-11
2	43283	biopolymer metabolic process	39/48 genes, 81.2%	1050/2464 genes, 42.6%	3.22E-06
5	44260	cellular macromolecule metabolic process	31/48 genes, 64.6%	786/2464 genes, 31.9%	0.00024
5	44267	cellular protein metabolic process	30/48 genes, 62.5%	756/2464 genes, 30.7%	0.00038
5	19538	protein metabolic process	30/48 genes, 62.5%	781/2464 genes, 31.7%	0.00081

It continues on next page

Table 1 – continued from previous page

Rep	GOID	GO-term	Frequency		*P-value*
			Cluster	Background	
2	43170	macromolecule metabolic process	41/48 genes, 85.4%	1417/2464 genes, 57.5%	0.00254
	**Cluster**	15			
1	79	regulation of cyclin-dependent protein kinase activity	3/12 genes, 25.0%	18/2464 genes, 0.7%	0.00505
1	43549	regulation of kinase activity	3/12 genes, 25.0%	20/2464 genes, 0.8%	0.00702
1	45859	regulation of protein kinase activity	3/12 genes, 25.0%	20/2464 genes, 0.8%	0.00702
1	51338	regulation of transferase activity	3/12 genes, 25.0%	21/2464 genes, 0.9%	0.00816
	**Cluster**	16			
1	43413	biopolymer glycosylation	7/15 genes, 46.7%	53/2464 genes, 2.2%	3.76E-07
1	6486	protein amino acid glycosylation	7/15 genes, 46.7%	53/2464 genes, 2.2%	3.76E-07
1	9100	glycoprotein metabolic process	7/15 genes, 46.7%	54/2464 genes, 2.2%	4.31E-07
1	9101	glycoprotein biosynthetic process	7/15 genes, 46.7%	54/2464 genes, 2.2%	4.31E-07
1	6464	protein modification process	9/15 genes, 60.0%	275/2464 genes, 11.2%	0.0003
1	43412	biopolymer modification	9/15 genes, 60.0%	301/2464 genes, 12.2%	0.00065
1	6493	protein amino acid O-linked glycosylation	3/15 genes, 20.0%	12/2464 genes, 0.5%	0.00183
1	6487	protein amino acid N-linked glycosylation	4/15 genes, 26.7%	41/2464 genes, 1.7%	0.00371
6	9059	macromolecule biosynthetic process	9/15 genes, 60.0%	391/2464 genes, 15.9%	0.00563
	**Cluster**	18			
2	19941	modification-dependent protein catabolic process	9/17 genes, 52.9%	93/2464 genes, 3.8%	1.60E-07
2	51603	proteolysis involved in cellular protein catabolic process	9/17 genes, 52.9%	93/2464 genes, 3.8%	1.60E-07
2	6511	ubiquitin-dependent protein catabolic process	9/17 genes, 52.9%	93/2464 genes, 3.8%	1.60E-07
2	43632	modification-dependent macromolecule catabolic process	9/17 genes, 52.9%	96/2464 genes, 3.9%	2.13E-07
2	44257	cellular protein catabolic process	9/17 genes, 52.9%	98/2464 genes, 4.0%	2.58E-07
2	30163	protein catabolic process	9/17 genes, 52.9%	104/2464 genes, 4.2%	4.42E-07
2	6508	proteolysis	9/17 genes, 52.9%	107/2464 genes, 4.3%	5.72E-07
2	43285	biopolymer catabolic process	9/17 genes, 52.9%	168/2464 genes, 6.8%	3.12E-05
2	44265	cellular macromolecule catabolic process	9/17 genes, 52.9%	182/2464 genes, 7.4%	6.25E-05
2	9057	macromolecule catabolic process	9/17 genes, 52.9%	203/2464 genes, 8.2%	0.00016
3	44248	cellular catabolic process	9/17 genes, 52.9%	258/2464 genes, 10.5%	0.00121
3	9056	catabolic process	9/17 genes, 52.9%	264/2464 genes, 10.7%	0.00146
	**Cluster**	19			
6	6412	translation	20/55 genes, 36.4%	293/2464 genes, 11.9%	0.00022
2	8643	carbohydrate transport	6/55 genes, 10.9%	25/2464 genes, 1.0%	0.0015
	**Cluster**	20			
1	19236	response to pheromone	9/16 genes, 56.2%	30/2464 genes, 1.2%	1.39E-12
1	19953	sexual reproduction	10/16 genes, 62.5%	48/2464 genes, 1.9%	1.76E-12
1	746	conjugation	10/16 genes, 62.5%	48/2464 genes, 1.9%	1.76E-12
1	747	conjugation with cellular fusion	10/16 genes, 62.5%	48/2464 genes, 1.9%	1.76E-12
1	51704	multi-organism process	10/16 genes, 62.5%	56/2464 genes, 2.3%	9.44E-12
1	749	response to pheromone during conjugation with cellular fusion	7/16 genes, 43.8%	24/2464 genes, 1.0%	2.83E-09
1	3	reproduction	11/16 genes, 68.8%	151/2464 genes, 6.1%	8.82E-09
1	31137	regulation of conjugation with cellular fusion	5/16 genes, 31.2%	10/2464 genes, 0.4%	1.17E-07
1	32005	signal transduction during conjugation with cellular fusion	5/16 genes, 31.2%	10/2464 genes, 0.4%	1.17E-07
1	46999	regulation of conjugation	5/16 genes, 31.2%	10/2464 genes, 0.4%	1.17E-07
1	750	pheromone-dependent signal transduction during conjugation with cellular fusion	5/16 genes, 31.2%	10/2464 genes, 0.4%	1.17E-07
1	7186	G-protein coupled receptor protein signaling pathway	5/16 genes, 31.2%	11/2464 genes, 0.4%	2.14E-07
1	7166	cell surface receptor linked signal transduction	5/16 genes, 31.2%	15/2464 genes, 0.6%	1.37E-06
1	42221	response to chemical stimulus	9/16 genes, 56.2%	172/2464 genes, 7.0%	1.96E-05
1	50896	response to stimulus	11/16 genes, 68.8%	406/2464 genes, 16.5%	0.00035
1	9968	negative regulation of signal transduction	3/16 genes, 18.8%	7/2464 genes, 0.3%	0.00064
1	7165	signal transduction	6/16 genes, 37.5%	87/2464 genes, 3.5%	0.00081
1	9966	regulation of signal transduction	3/16 genes, 18.8%	9/2464 genes, 0.4%	0.00151
2	7154	cell communication	6/16 genes, 37.5%	107/2464 genes, 4.3%	0.00269

It continues on next page

Rep	GOID	GO-term	Frequency		P-value
			Cluster	Background	
1	50794	regulation of cellular process	10/16 genes, 62.5%	436/2464 genes, 17.7%	0.00649
1	50789	regulation of biological process	10/16 genes, 62.5%	446/2464 genes, 18.1%	0.00796
	**Cluster**	21			
6	6412	translation	22/67 genes, 32.8%	293/2464 genes, 11.9%	0.00053
6	9059	macromolecule biosynthetic process	24/67 genes, 35.8%	391/2464 genes, 15.9%	0.0058
	**Cluster**	22			
1	255	allantoin metabolic process	5/19 genes, 26.3%	7/2464 genes, 0.3%	2.34E-08
1	256	allantoin catabolic process	5/19 genes, 26.3%	7/2464 genes, 0.3%	2.34E-08
1	46700	heterocycle catabolic process	5/19 genes, 26.3%	7/2464 genes, 0.3%	2.34E-08
2	6807	nitrogen compound metabolic process	12/19 genes, 63.2%	189/2464 genes, 7.7%	6.77E-08
1	19740	nitrogen utilization	4/19 genes, 21.1%	12/2464 genes, 0.5%	8.79E-05
2	9308	amine metabolic process	8/19 genes, 42.1%	175/2464 genes, 7.1%	0.00155
1	46483	heterocycle metabolic process	5/19 genes, 26.3%	63/2464 genes, 2.6%	0.006
	**Cluster**	24			
1	44262	cellular carbohydrate metabolic process	6/11 genes, 54.5%	120/2464 genes, 4.9%	0.00016
1	5975	carbohydrate metabolic process	6/11 genes, 54.5%	130/2464 genes, 5.3%	0.00026
2	32787	monocarboxylic acid metabolic process	5/11 genes, 45.5%	84/2464 genes, 3.4%	0.00058
2	19752	carboxylic acid metabolic process	7/11 genes, 63.6%	244/2464 genes, 9.9%	0.00072
2	6082	organic acid metabolic process	7/11 genes, 63.6%	244/2464 genes, 9.9%	0.00072
1	6094	gluconeogenesis	3/11 genes, 27.3%	21/2464 genes, 0.9%	0.00304
1	46487	glyoxylate metabolic process	2/11 genes, 18.2%	4/2464 genes, 0.2%	0.0039
1	6097	glyoxylate cycle	2/11 genes, 18.2%	4/2464 genes, 0.2%	0.0039
1	19319	hexose biosynthetic process	3/11 genes, 27.3%	23/2464 genes, 0.9%	0.00402
1	46364	monosaccharide biosynthetic process	3/11 genes, 27.3%	23/2464 genes, 0.9%	0.00402
2	46165	alcohol biosynthetic process	3/11 genes, 27.3%	25/2464 genes, 1.0%	0.0052
2	6090	pyruvate metabolic process	3/11 genes, 27.3%	30/2464 genes, 1.2%	0.00907
	**Cluster**	25			
1	6096	glycolysis	15/239 genes, 6.3%	19/2464 genes, 0.8%	6.60E-10
1	6007	glucose catabolic process	17/239 genes, 7.1%	28/2464 genes, 1.1%	1.62E-08
1	19320	hexose catabolic process	17/239 genes, 7.1%	30/2464 genes, 1.2%	7.58E-08
1	46365	monosaccharide catabolic process	17/239 genes, 7.1%	31/2464 genes, 1.3%	1.53E-07
2	6066	alcohol metabolic process	31/239 genes, 13.0%	97/2464 genes, 3.9%	2.39E-07
1	46164	alcohol catabolic process	17/239 genes, 7.1%	33/2464 genes, 1.3%	5.64E-07
1	16052	carbohydrate catabolic process	17/239 genes, 7.1%	39/2464 genes, 1.6%	1.44E-05
1	44275	cellular carbohydrate catabolic process	17/239 genes, 7.1%	39/2464 genes, 1.6%	1.44E-05
1	34308	monohydric alcohol metabolic process	7/239 genes, 2.9%	7/2464 genes, 0.3%	4.30E-05
1	6067	ethanol metabolic process	7/239 genes, 2.9%	7/2464 genes, 0.3%	4.30E-05
1	19318	hexose metabolic process	20/239 genes, 8.4%	56/2464 genes, 2.3%	4.70E-05
1	5996	monosaccharide metabolic process	20/239 genes, 8.4%	58/2464 genes, 2.4%	9.23E-05
1	6006	glucose metabolic process	18/239 genes, 7.5%	49/2464 genes, 2.0%	0.00013
2	32787	monocarboxylic acid metabolic process	24/239 genes, 10.0%	84/2464 genes, 3.4%	0.00029
2	6090	pyruvate metabolic process	13/239 genes, 5.4%	30/2464 genes, 1.2%	0.00075
1	6113	fermentation	7/239 genes, 2.9%	10/2464 genes, 0.4%	0.00399
2	46165	alcohol biosynthetic process	11/239 genes, 4.6%	25/2464 genes, 1.0%	0.0043
1	19655	glucose catabolic process to ethanol	5/239 genes, 2.1%	5/2464 genes, 0.2%	0.00478
1	19660	glycolytic fermentation	5/239 genes, 2.1%	5/2464 genes, 0.2%	0.00478
1	6766	vitamin metabolic process	15/239 genes, 6.3%	45/2464 genes, 1.8%	0.00572
1	6767	water-soluble vitamin metabolic process	15/239 genes, 6.3%	45/2464 genes, 1.8%	0.00572
	**Cluster**	26			
2	6457	protein folding	3/4 genes, 75.0%	55/2464 genes, 2.2%	0.0005
1	7010	cytoskeleton organization and biogenesis	3/4 genes, 75.0%	122/2464 genes, 5.0%	0.00548
	**Cluster**	28			

It continues on next page

Table 1 – continued from previous page

Rep	GOID	GO-term	Frequency		*P-value*
			Cluster	Background	
2	6457	protein folding	15/30 genes, 50.0%	55/2464 genes, 2.2%	3.03E-16
1	6616	SRP-dependent cotranslational protein targeting to membrane, translocation	5/30 genes, 16.7%	8/2464 genes, 0.3%	1.17E-06
1	42026	protein refolding	5/30 genes, 16.7%	9/2464 genes, 0.4%	2.62E-06
1	65002	intracellular protein transport across a membrane	5/30 genes, 16.7%	12/2464 genes, 0.5%	1.60E-05
2	45047	protein targeting to ER	6/30 genes, 20.0%	24/2464 genes, 1.0%	2.53E-05
1	6614	SRP-dependent cotranslational protein targeting to membrane	5/30 genes, 16.7%	16/2464 genes, 0.6%	8.57E-05
1	6613	cotranslational protein targeting to membrane	5/30 genes, 16.7%	17/2464 genes, 0.7%	0.00012
5	44267	cellular protein metabolic process	22/30 genes, 73.3%	756/2464 genes, 30.7%	0.00018
5	19538	protein metabolic process	22/30 genes, 73.3%	781/2464 genes, 31.7%	0.00034
5	44260	cellular macromolecule metabolic process	22/30 genes, 73.3%	786/2464 genes, 31.9%	0.00039
1	6612	protein targeting to membrane	5/30 genes, 16.7%	29/2464 genes, 1.2%	0.00209
1	15031	protein transport	9/30 genes, 30.0%	157/2464 genes, 6.4%	0.00705
	**Cluster**	30			
1	6888	ER to Golgi vesicle-mediated transport	3/3 genes, 100.0%	43/2464 genes, 1.7%	6.93E-05
1	48193	Golgi vesicle transport	3/3 genes, 100.0%	93/2464 genes, 3.8%	0.00073
1	45045	secretory pathway	3/3 genes, 100.0%	137/2464 genes, 5.6%	0.00236
1	32940	secretion by cell	3/3 genes, 100.0%	140/2464 genes, 5.7%	0.00252
1	46903	secretion	3/3 genes, 100.0%	140/2464 genes, 5.7%	0.00252
2	16192	vesicle-mediated transport	3/3 genes, 100.0%	174/2464 genes, 7.1%	0.00485
	**Cluster**	31			
1	6696	ergosterol biosynthetic process	5/13 genes, 38.5%	22/2464 genes, 0.9%	2.44E-06
1	8204	ergosterol metabolic process	5/13 genes, 38.5%	22/2464 genes, 0.9%	2.44E-06
1	16126	sterol biosynthetic process	5/13 genes, 38.5%	23/2464 genes, 0.9%	3.11E-06
1	6694	steroid biosynthetic process	5/13 genes, 38.5%	23/2464 genes, 0.9%	3.11E-06
1	16125	sterol metabolic process	5/13 genes, 38.5%	27/2464 genes, 1.1%	7.39E-06
1	8202	steroid metabolic process	5/13 genes, 38.5%	27/2464 genes, 1.1%	7.39E-06
1	8610	lipid biosynthetic process	5/13 genes, 38.5%	72/2464 genes, 2.9%	0.00113
2	6066	alcohol metabolic process	5/13 genes, 38.5%	97/2464 genes, 3.9%	0.00487
1	6555	methionine metabolic process	3/13 genes, 23.1%	19/2464 genes, 0.8%	0.00604
	**Cluster**	32			
3	42254	ribosome biogenesis and assembly	55/161 genes, 34.2%	145/2464 genes, 5.9%	1.11E-28
3	22613	ribonucleoprotein complex biogenesis and assembly	63/161 genes, 39.1%	197/2464 genes, 8.0%	1.44E-28
1	16070	RNA metabolic process	88/161 genes, 54.7%	584/2464 genes, 23.7%	6.48E-16
2	6364	rRNA processing	30/161 genes, 18.6%	73/2464 genes, 3.0%	2.24E-15
2	16072	rRNA metabolic process	30/161 genes, 18.6%	74/2464 genes, 3.0%	3.57E-15
1	6396	RNA processing	49/161 genes, 30.4%	204/2464 genes, 8.3%	4.60E-15
1	6139	nucleobase, nucleoside, nucleotide and nucleic acid metabolic process	102/161 genes, 63.4%	835/2464 genes, 33.9%	1.11E-12
1	42273	ribosomal large subunit biogenesis and assembly	15/161 genes, 9.3%	32/2464 genes, 1.3%	8.18E-08
4	10467	gene expression	91/161 genes, 56.5%	835/2464 genes, 33.9%	4.02E-07
2	43283	biopolymer metabolic process	101/161 genes, 62.7%	1050/2464 genes, 42.6%	3.68E-05
2	8152	metabolic process	146/161 genes, 90.7%	1844/2464 genes, 74.8%	6.09E-05
1	466	maturation of 5.8S rRNA from tricistronic rRNA transcript (SSU-rRNA, 5.8S rRNA, LSU-rRNA)	7/161 genes, 4.3%	9/2464 genes, 0.4%	6.18E-05
1	9451	RNA modification	11/161 genes, 6.8%	27/2464 genes, 1.1%	0.00015
1	6401	RNA catabolic process	14/161 genes, 8.7%	45/2464 genes, 1.8%	0.00018
1	460	maturation of 5.8S rRNA	7/161 genes, 4.3%	10/2464 genes, 0.4%	0.00019
1	463	maturation of LSU-rRNA from tricistronic rRNA transcript (SSU-rRNA, 5.8S rRNA, LSU-rRNA)	6/161 genes, 3.7%	7/2464 genes, 0.3%	0.0002
1	470	maturation of LSU-rRNA	6/161 genes, 3.7%	7/2464 genes, 0.3%	0.0002
2	44238	primary metabolic process	138/161 genes, 85.7%	1713/2464 genes, 69.5%	0.00032
1	22618	protein-RNA complex assembly	20/161 genes, 12.4%	92/2464 genes, 3.7%	0.00037
2	44237	cellular metabolic process	142/161 genes, 88.2%	1798/2464 genes, 73.0%	0.00046

It continues on next page

Table 1 – continued from previous page

Rep	GOID	GO-term	Frequency		*P-value*
			**Cluster**	**Background**	
1	6996	organelle organization and biogenesis	75/161 genes, 46.6%	727/2464 genes, 29.5%	0.00069
1	43094	metabolic compound salvage	7/161 genes, 4.3%	12/2464 genes, 0.5%	0.00115
2	16043	cellular component organization and biogenesis	102/161 genes, 63.4%	1137/2464 genes, 46.1%	0.00181
1	6400	tRNA modification	8/161 genes, 5.0%	18/2464 genes, 0.7%	0.00299
1	6402	mRNA catabolic process	11/161 genes, 6.8%	35/2464 genes, 1.4%	0.00299
1	43101	purine salvage	5/161 genes, 3.1%	7/2464 genes, 0.3%	0.00906
1	65003	macromolecular complex assembly	31/161 genes, 19.3%	223/2464 genes, 9.1%	0.00989
	**Cluster**	34			
1	6979	response to oxidative stress	3/9 genes, 33.3%	31/2464 genes, 1.3%	0.00763
	**Cluster**	35			
6	6412	translation	42/64 genes, 65.6%	293/2464 genes, 11.9%	7.33E-23
6	9059	macromolecule biosynthetic process	42/64 genes, 65.6%	391/2464 genes, 15.9%	1.11E-17
3	44249	cellular biosynthetic process	46/64 genes, 71.9%	523/2464 genes, 21.2%	1.66E-16
3	9058	biosynthetic process	46/64 genes, 71.9%	665/2464 genes, 27.0%	4.31E-12
5	44267	cellular protein metabolic process	48/64 genes, 75.0%	756/2464 genes, 30.7%	1.79E-11
5	19538	protein metabolic process	48/64 genes, 75.0%	781/2464 genes, 31.7%	7.15E-11
5	44260	cellular macromolecule metabolic process	48/64 genes, 75.0%	786/2464 genes, 31.9%	9.36E-11
4	10467	gene expression	49/64 genes, 76.6%	835/2464 genes, 33.9%	1.84E-10
2	43170	macromolecule metabolic process	56/64 genes, 87.5%	1417/2464 genes, 57.5%	1.52E-05
2	44238	primary metabolic process	60/64 genes, 93.8%	1713/2464 genes, 69.5%	0.00018
2	44237	cellular metabolic process	60/64 genes, 93.8%	1798/2464 genes, 73.0%	0.00215
2	8152	metabolic process	60/64 genes, 93.8%	1844/2464 genes, 74.8%	0.00767
	**Cluster**	36			
2	16192	vesicle-mediated transport	6/11 genes, 54.5%	174/2464 genes, 7.1%	0.00125
	**Cluster**	39			
6	6412	translation	11/15 genes, 73.3%	293/2464 genes, 11.9%	2.48E-06
6	9059	macromolecule biosynthetic process	11/15 genes, 73.3%	391/2464 genes, 15.9%	5.27E-05
3	44249	cellular biosynthetic process	12/15 genes, 80.0%	523/2464 genes, 21.2%	9.07E-05
4	10467	gene expression	14/15 genes, 93.3%	835/2464 genes, 33.9%	0.00013
3	9058	biosynthetic process	12/15 genes, 80.0%	665/2464 genes, 27.0%	0.00136
5	44267	cellular protein metabolic process	12/15 genes, 80.0%	756/2464 genes, 30.7%	0.00557
5	19538	protein metabolic process	12/15 genes, 80.0%	781/2464 genes, 31.7%	0.00794
5	44260	cellular macromolecule metabolic process	12/15 genes, 80.0%	786/2464 genes, 31.9%	0.00851
	**Cluster**	42			
6	6412	translation	11/12 genes, 91.7%	293/2464 genes, 11.9%	1.95E-08
3	44249	cellular biosynthetic process	12/12 genes, 100.0%	523/2464 genes, 21.2%	2.42E-07
6	9059	macromolecule biosynthetic process	11/12 genes, 91.7%	391/2464 genes, 15.9%	4.69E-07
3	9058	biosynthetic process	12/12 genes, 100.0%	665/2464 genes, 27.0%	4.44E-06
1	6450	regulation of translational fidelity	3/12 genes, 25.0%	10/2464 genes, 0.4%	0.00033
5	44267	cellular protein metabolic process	11/12 genes, 91.7%	756/2464 genes, 30.7%	0.0006
5	19538	protein metabolic process	11/12 genes, 91.7%	781/2464 genes, 31.7%	0.00085
5	44260	cellular macromolecule metabolic process	11/12 genes, 91.7%	786/2464 genes, 31.9%	0.0009
4	10467	gene expression	11/12 genes, 91.7%	835/2464 genes, 33.9%	0.00172
	**Cluster**	43			
3	22613	ribonucleoprotein complex biogenesis and assembly	13/17 genes, 76.5%	197/2464 genes, 8.0%	3.68E-10
3	42254	ribosome biogenesis and assembly	10/17 genes, 58.8%	145/2464 genes, 5.9%	2.75E-07
2	6364	rRNA processing	7/17 genes, 41.2%	73/2464 genes, 3.0%	1.26E-05
2	16072	rRNA metabolic process	7/17 genes, 41.2%	74/2464 genes, 3.0%	1.39E-05
2	16043	cellular component organization and biogenesis	16/17 genes, 94.1%	1137/2464 genes, 46.1%	0.00212
	**Cluster**	44			
1	9082	branched chain family amino acid biosynthetic process	4/30 genes, 13.3%	11/2464 genes, 0.4%	0.00055
1	9081	branched chain family amino acid metabolic process	4/30 genes, 13.3%	13/2464 genes, 0.5%	0.00117

It continues on next page

Table 1 – continued from previous page

Rep	GOID	GO-term	Frequency		*P-value*
			**Cluster**	**Background**	
3	42254	ribosome biogenesis and assembly	9/30 genes, 30.0%	145/2464 genes, 5.9%	0.00323
3	22613	ribonucleoprotein complex biogenesis and assembly	10/30 genes, 33.3%	197/2464 genes, 8.0%	0.00612
	**Cluster**	46			
1	6267	pre-replicative complex assembly	4/12 genes, 33.3%	14/2464 genes, 0.6%	1.70E-05
1	84	S phase of mitotic cell cycle	4/12 genes, 33.3%	15/2464 genes, 0.6%	2.31E-05
1	51320	S phase	4/12 genes, 33.3%	17/2464 genes, 0.7%	4.01E-05
1	22616	DNA strand elongation	4/12 genes, 33.3%	30/2464 genes, 1.2%	0.00045
1	6271	DNA strand elongation during DNA replication	4/12 genes, 33.3%	30/2464 genes, 1.2%	0.00045
1	6261	DNA-dependent DNA replication	5/12 genes, 41.7%	80/2464 genes, 3.2%	0.00114
2	6260	DNA replication	5/12 genes, 41.7%	96/2464 genes, 3.9%	0.00279
1	43623	cellular protein complex assembly	4/12 genes, 33.3%	48/2464 genes, 1.9%	0.00303
	**Cluster**	47			
1	910	cytokinesis	7/15 genes, 46.7%	64/2464 genes, 2.6%	2.18E-06
1	51301	cell division	7/15 genes, 46.7%	82/2464 genes, 3.3%	1.27E-05
1	32506	cytokinetic process	5/15 genes, 33.3%	55/2464 genes, 2.2%	0.00082
1	916	contractile ring contraction involved in cytokinesis	2/15 genes, 13.3%	3/2464 genes, 0.1%	0.00724
2	22402	cell cycle process	7/15 genes, 46.7%	210/2464 genes, 8.5%	0.00737
	**Cluster**	51			
1	43562	cellular response to nitrogen levels	4/4 genes, 100.0%	5/2464 genes, 0.2%	1.14E-10
1	6530	asparagine catabolic process	4/4 genes, 100.0%	5/2464 genes, 0.2%	1.14E-10
1	6995	cellular response to nitrogen starvation	4/4 genes, 100.0%	5/2464 genes, 0.2%	1.14E-10
1	9068	aspartate family amino acid catabolic process	4/4 genes, 100.0%	6/2464 genes, 0.2%	3.42E-10
1	6528	asparagine metabolic process	4/4 genes, 100.0%	8/2464 genes, 0.3%	1.59E-09
1	9065	glutamine family amino acid catabolic process	4/4 genes, 100.0%	11/2464 genes, 0.4%	7.53E-09
1	42594	response to starvation	4/4 genes, 100.0%	15/2464 genes, 0.6%	3.11E-08
1	9267	cellular response to starvation	4/4 genes, 100.0%	15/2464 genes, 0.6%	3.11E-08
1	33554	cellular response to stress	4/4 genes, 100.0%	18/2464 genes, 0.7%	6.99E-08
1	9063	amino acid catabolic process	4/4 genes, 100.0%	19/2464 genes, 0.8%	8.85E-08
1	44270	nitrogen compound catabolic process	4/4 genes, 100.0%	21/2464 genes, 0.9%	1.36E-07
1	9310	amine catabolic process	4/4 genes, 100.0%	21/2464 genes, 0.9%	1.36E-07
1	31668	cellular response to extracellular stimulus	4/4 genes, 100.0%	23/2464 genes, 0.9%	2.02E-07
1	31669	cellular response to nutrient levels	4/4 genes, 100.0%	23/2464 genes, 0.9%	2.02E-07
1	31667	response to nutrient levels	4/4 genes, 100.0%	24/2464 genes, 1.0%	2.42E-07
1	9605	response to external stimulus	4/4 genes, 100.0%	24/2464 genes, 1.0%	2.42E-07
1	9991	response to extracellular stimulus	4/4 genes, 100.0%	24/2464 genes, 1.0%	2.42E-07
1	51716	cellular response to stimulus	4/4 genes, 100.0%	26/2464 genes, 1.1%	3.41E-07
1	9064	glutamine family amino acid metabolic process	4/4 genes, 100.0%	38/2464 genes, 1.5%	1.68E-06
1	9066	aspartate family amino acid metabolic process	4/4 genes, 100.0%	38/2464 genes, 1.5%	1.68E-06
2	7154	cell communication	4/4 genes, 100.0%	107/2464 genes, 4.3%	0.00012
1	6520	amino acid metabolic process	4/4 genes, 100.0%	157/2464 genes, 6.4%	0.00056
1	6519	amino acid and derivative metabolic process	4/4 genes, 100.0%	167/2464 genes, 6.8%	0.00071
2	9308	amine metabolic process	4/4 genes, 100.0%	175/2464 genes, 7.1%	0.00086
2	6807	nitrogen compound metabolic process	4/4 genes, 100.0%	189/2464 genes, 7.7%	0.00118
2	19752	carboxylic acid metabolic process	4/4 genes, 100.0%	244/2464 genes, 9.9%	0.00329
2	6082	organic acid metabolic process	4/4 genes, 100.0%	244/2464 genes, 9.9%	0.00329
3	44248	cellular catabolic process	4/4 genes, 100.0%	258/2464 genes, 10.5%	0.00412
3	9056	catabolic process	4/4 genes, 100.0%	264/2464 genes, 10.7%	0.00452
1	6950	response to stress	4/4 genes, 100.0%	282/2464 genes, 11.4%	0.00589
