# Supplementary material for: QAPgrid: A Two Level QAP-Based Approach for Large-Scale Data Analysis and Visualization
Source: PLoS One. 2011 Jan 18;6(1):e14468. doi: 10.1371/journal.pone.0014468 (PMC3022583; doi:10.1371/journal.pone.0014468)
Supplement: File S5 — Clustering of genes (yeast dataset) as in Figures 13, 14 and 15 (at a higher resolution). (1.53 MB PDF) [file pone.0014468.s005.pdf]

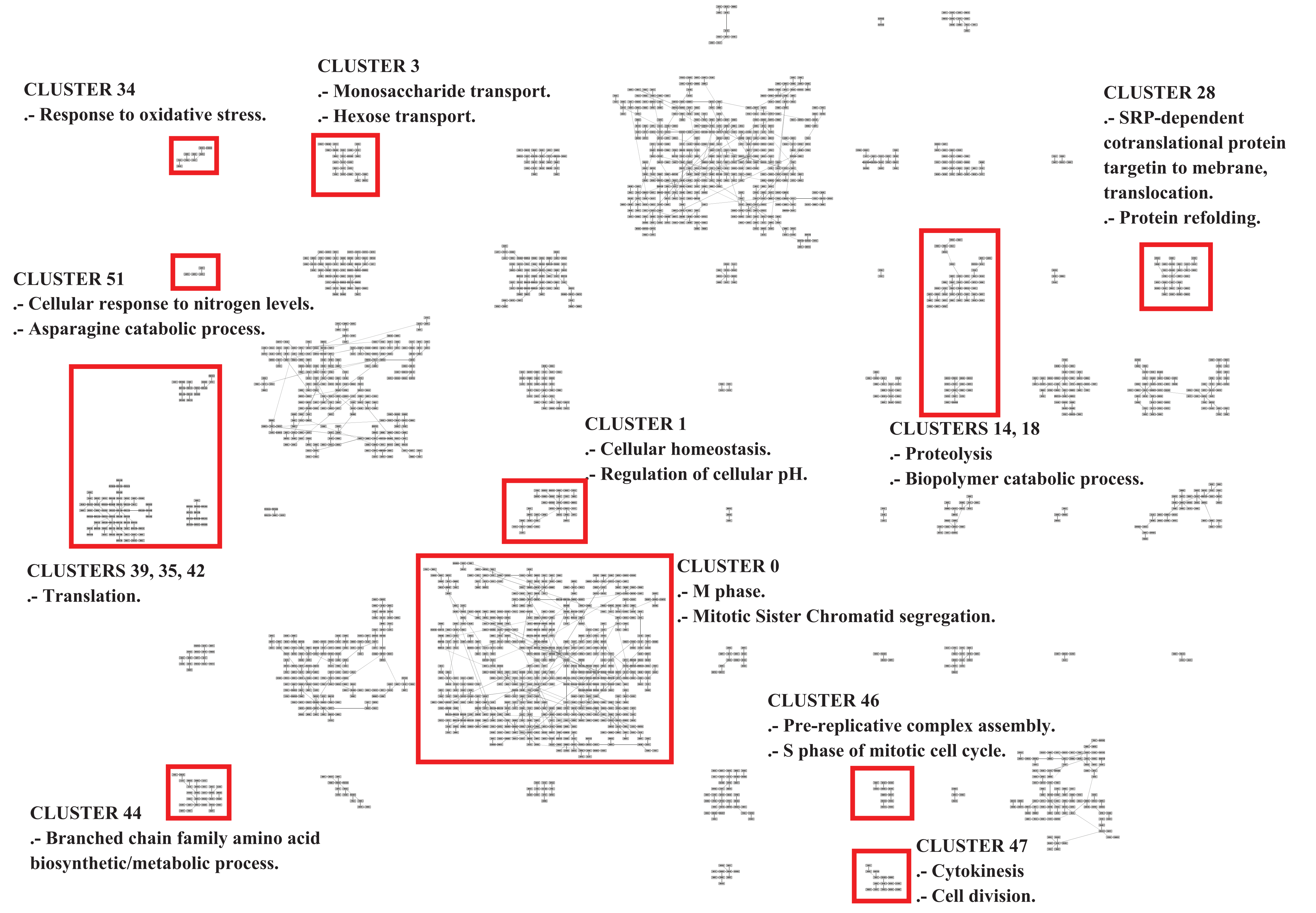
CLUSTER 34
.- Response to oxidative stress.
CLUSTER 3
.- Monosaccharide transport.
.- Hexose transport.
CLUSTER 28
.- SRP-dependent
cotranslational protein
targetin to mebrane,
translocation.
.- Protein refolding.
CLUSTER 51
.- Cellular response to nitrogen levels.
.- Asparagine catabolic process.
CLUSTER 1
.- Cellular homeostasis.
.- Regulation of cellular pH.
CLUSTERS 14, 18
.- Proteolysis
.- Biopolymer catabolic process.
CLUSTERS 39, 35, 42
.- Translation.
CLUSTER 0
.- M phase.
.- Mitotic Sister Chromatid segregation.
CLUSTER 46
.- Pre-replicative complex assembly.
.- S phase of mitotic cell cycle.
CLUSTER 44
.- Branched chain family amino acid
biosynthetic/metabolic process.
CLUSTER 47
.- Cytokinesis
.- Cell division.

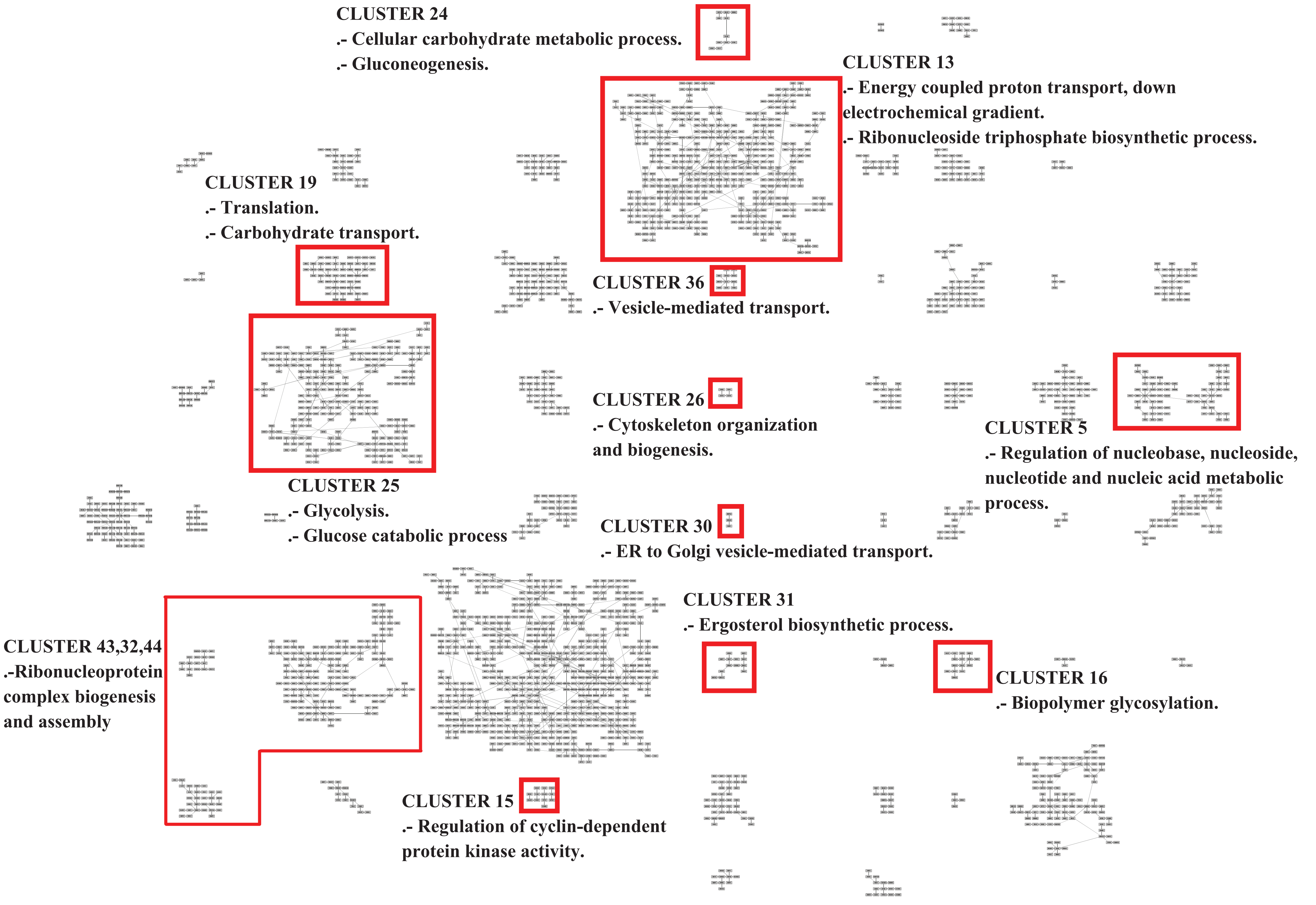
CLUSTER 24
.- Cellular carbohydrate metabolic process.
.- Gluconeogenesis.
CLUSTER 13
.- Energy coupled proton transport, down electrochemical gradient.
.- Ribonucleoside triphosphate biosynthetic process.
CLUSTER 19
.- Translation.
.- Carbohydrate transport.
CLUSTER 36
.- Vesicle-mediated transport.
CLUSTER 26
.- Cytoskeleton organization and biogenesis.
CLUSTER 5
.- Regulation of nucleobase, nucleoside, nucleotide and nucleic acid metabolic process.
CLUSTER 25
.- Glycolysis.
.- Glucose catabolic process
CLUSTER 30
.- ER to Golgi vesicle-mediated transport.
CLUSTER 31
.- Ergosterol biosynthetic process.
CLUSTER 43,32,44
.-Ribonucleoprotein complex biogenesis and assembly
CLUSTER 16
.- Biopolymer glycosylation.
CLUSTER 15
.- Regulation of cyclin-dependent protein kinase activity.

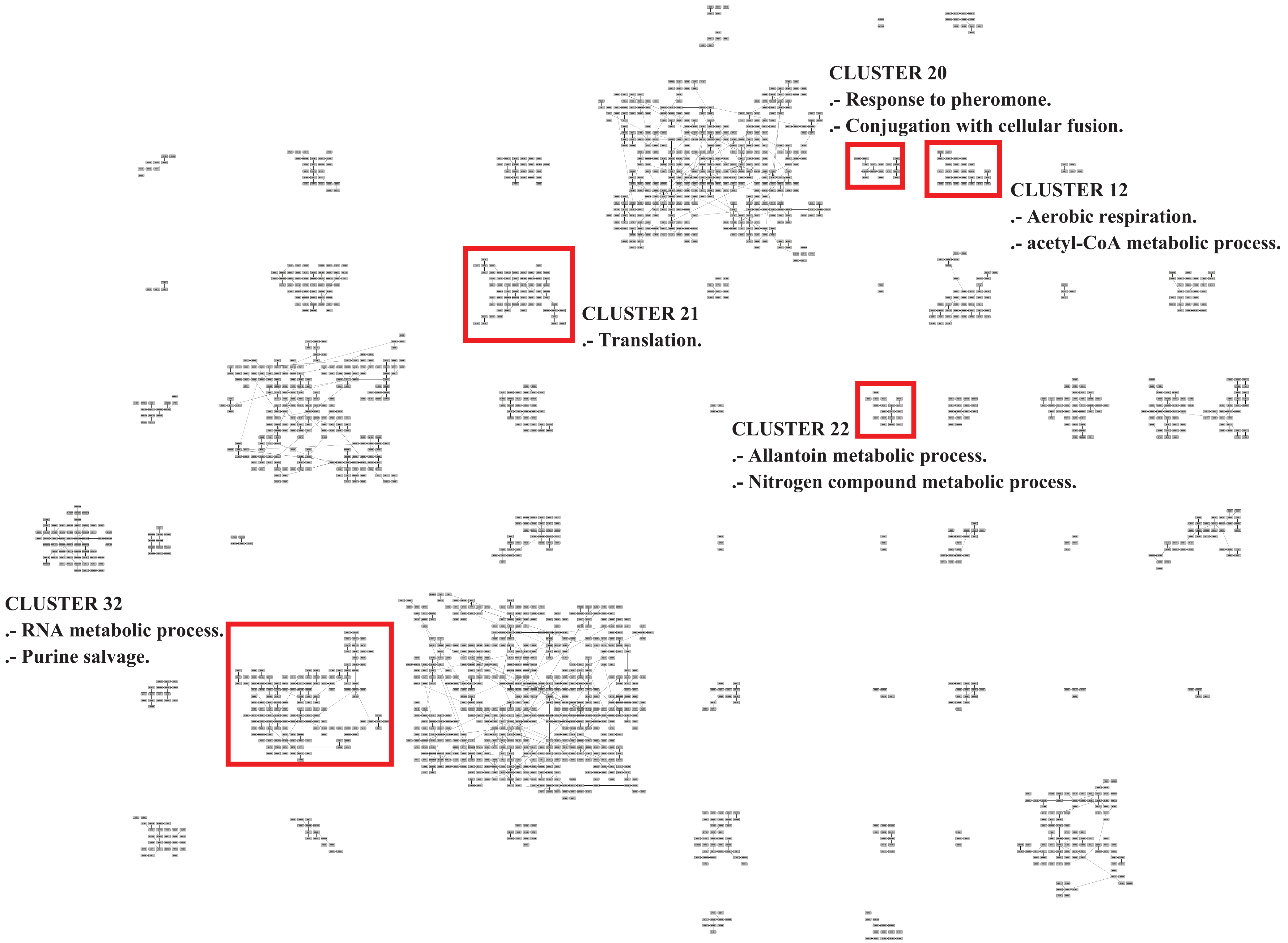

CLUSTER 20
.- Response to pheromone.
.- Conjugation with cellular fusion.
CLUSTER 12
.- Aerobic respiration.
.- acetyl-CoA metabolic process.
CLUSTER 21
.- Translation.
CLUSTER 22
.- Allantoin metabolic process.
.- Nitrogen compound metabolic process.
CLUSTER 32
.- RNA metabolic process.
.- Purine salvage.
